# Vasculotide reduces endothelial permeability and tumor cell extravasation in the absence of binding to or agonistic activation of Tie2

**DOI:** 10.15252/emmm.201404193

**Published:** 2015-04-07

**Authors:** Florence TH Wu, Christina R Lee, Elena Bogdanovic, Aaron Prodeus, Jean Gariépy, Robert S Kerbel

**Affiliations:** 1 Department of Medical Biophysics, University of TorontoToronto, ON, Canada; 2 Biological Sciences Platform, Sunnybrook Research InstituteToronto, ON, Canada; 3 Physical Sciences Platform, Sunnybrook Research InstituteToronto, ON, Canada

**Keywords:** angiopoietin, metastasis, Tie2, tumor cell extravasation, vascular permeability

## Abstract

Angiopoietin-1 (Ang1) activation of Tie2 receptors on endothelial cells (ECs) reduces adhesion by tumor cells (TCs) and limits junctional permeability to TC diapedesis. We hypothesized that systemic therapy with Vasculotide (VT)—a purported Ang1 mimetic, Tie2 agonist—can reduce the extravasation of potentially metastatic circulating TCs by similarly stabilizing the host vasculature. *In vitro,* VT and Ang1 treatments impeded endothelial hypermeability and the transendothelial migration of MDA-MB-231•LM2-4 (breast), HT29 (colon), or SN12 (renal) cancer cells to varying degrees. In mice, VT treatment inhibited the transit of TCs through the pulmonary endothelium, but not the hepatic or lymphatic endothelium. In the *in vivo* LM2-4 model, VT monotherapy had no effect on primary tumors, but significantly delayed distant metastatic dissemination to the lungs. In the post-surgical adjuvant treatment setting, VT therapeutically complemented sunitinib therapy, an anti-angiogenic tyrosine kinase inhibitor which limited the local growth of residual disease. Unexpectedly, detailed investigations into the putative mechanism of action of VT revealed no evidence of Tie2 agonism or Tie2 binding; alternative mechanisms have yet to be determined.

## Introduction

Metastatic disease—as opposed to primary tumors—accounts for 90% of cancer-related mortality (Steeg, 2012). Most cancer drugs are selected from preclinical studies based on their potency at inhibiting primary tumor growth, and brought into clinical trials with the rationale that they will similarly inhibit the growth of metastases (Francia *et al*, 2011; Guerin *et al*, 2013). The inadequacy of this approach has been reviewed and editorials have highlighted the need for new anti-metastatic therapies that block not just the growth (progression) but also the spread or formation (incidence) of metastases (Steeg, 2012).

The metastatic process comprises a cascade of events (Talmadge & Fidler, 2010; Hanahan & Weinberg, 2011): stromal invasion from a localized tumor; intravasation of tumor cells (TCs); their systemic circulation and arrest in distant capillary beds; TC extravasation into the host organ parenchyma; and overt colonization as micrometastases grow into macrometastases. In clinically relevant settings, the temporal window of TC extravasation is asynchronous and wide: Primary tumors shed millions of TCs per gram of tumor into the blood circulation every day (Bockhorn *et al*, 2007), and metastases themselves can metastasize to tertiary sites or ‘self-seed’ back to primary sites (Comen *et al*, 2011). Surgical trauma associated with primary tumor resections can also sometimes paradoxically fuel metastatic spread—for example, by mechanically dislodging tumor cells into the circulation and by inducing the production/release of inflammatory and angiogenic cytokines that promote metastatic seeding and progression (Goldfarb & Ben-Eliyahu, 2006).

Tumor cell extravasation is regulated by many cytokines, including angiopoietin-1 (Ang1), a guardian of EC quiescence (Augustin *et al*, 2009; Huang *et al*, 2010), as well as angiopoietin-2 (Ang2) and vascular endothelial growth factor (VEGF), the cooperative initiator and driver of angiogenesis (Huang *et al*, 2010; Felcht *et al*, 2012). It was found that VEGF stimulates, Ang2 potentiates, while Ang1 counteracts the TNF-α/NF-κB-mediated EC surface expression of ICAM-1, VCAM-1, and E-selectin—which facilitate the adhesion and migration of TCs across the endothelium (Kim *et al*, 2001b; Fiedler *et al*, 2006; Miles *et al*, 2008; Huang *et al*, 2010). VEGF also stimulates, while Ang1 counteracts Src-mediated destabilization of paracellular VE-cadherin junctions and IP_3_/eNOS-mediated calcium influxes in ECs—which together cause vascular hyperpermeability and lowered resistance to TC diapedesis (Gamble *et al*, 2000; Gavard *et al*, 2008; Le Guelte *et al*, 2011; Koh, 2013). The angiopoietins have become targets of growing interest in the development of cancer therapeutics (Huang *et al*, 2010). Based on the above, both anti-Ang2 and pro-Ang1 strategies should have the potential to impede TC extravasation by reducing TC-EC adhesion and vascular permeability.

Preclinically, anti-Ang2 agents have largely been studied in the context of primary tumor growth, often demonstrating additive anti-angiogenic effects when combined with VEGF pathway inhibitors (Brown *et al*, 2010; Doppalapudi *et al*, 2010; Koh *et al*, 2010; Huang *et al*, 2011; Mazzieri *et al*, 2011; Schaefer *et al*, 2011; Holopainen *et al*, 2012; Leow *et al*, 2012). Some have further shown anti-metastatic activity preclinically through vascular-stabilizing systemic effects (Holopainen *et al*, 2012).

In the context of suppressing primary tumor growth by targeting the tumor vasculature, concurrent Ang1 inhibition was thought to augment the activity of Ang2-specific inhibition (Falcon *et al*, 2009; Coxon *et al*, 2010), but metastatic disease was not modeled in these preclinical studies. In the clinic, the dual Ang2/Ang1-neutralizing peptibody, trebananib (AMG386), recently failed to meet its secondary endpoint of overall survival, despite improving progression-free survival (PFS) in earlier primary endpoint analysis (Monk *et al*, 2014), when combined with paclitaxel for patients with recurrent platinum-sensitive ovarian cancer in the phase III TRINOVA-1 trial. Trebananib had also failed in phase II trials involving metastatic gastro-esophageal (Eatock *et al*, 2013), colorectal (Peeters *et al*, 2013), and clear-cell renal (Rini *et al*, 2012) carcinomas. This lack of clinical efficacy raises a question: Could concurrent Ang1 inhibition actually be compromising the anti-metastatic efficacy of Ang2-specific inhibition by destabilizing systemic blood vessels to promote distant metastatic spread, despite the expected additive benefit in local tumor growth suppression (Cascone & Heymach, 2012)? This is plausible, given the evidence that Ang1 inhibition can lead to systemic dysfunction of vessels in healthy tissues (Thomas *et al*, 2013).

On the other hand, preclinical evidence is conflicting on whether Ang1 overexpression by genetic approaches (Ahmad *et al*, 2001; Hawighorst *et al*, 2002; Tian *et al*, 2002; Machein *et al*, 2004; Holopainen *et al*, 2009; Hwang *et al*, 2009; Schulz *et al*, 2011) has positive or negative effects on tumor growth and metastasis. Recently, the subcutaneous administration of a recombinant Ang1 variant protein (‘Ang-F1-Fc-F1’, also called ‘BowAng1’) on its own had no effect on primary tumors, but as a concurrent treatment diminished the anti-angiogenic and anti-tumor efficacy of an anti-Ang2 antibody (Daly *et al*, 2013). This suggests that Ang1 supplementation may be counterproductive, even if not detrimental, in terms of controlling localized primary tumor growth; however, this aforementioned study did not model metastatic disease, where the impact may be fundamentally different (Guerin *et al*, 2013).

We hypothesize that systemic pro-Ang1 therapy can inhibit TC extravasation and metastatic spread, by reducing TC-EC adhesion and vascular permeability. In this study, we sought to test these hypotheses using Vasculotide (described below) as our candidate Ang1-mimetic vascular-stabilizing agent, with or without an anti-angiogenic VEGF pathway inhibitor, in *in vitro* models of tumor cell extravasation and *in vivo* models of metastasis.

The two angiopoietin ligands share the same cognate tyrosine kinase receptor, Tie2—with Ang1 being the main agonist, while Ang2 often acts as a competitive antagonist and sometimes as a partial agonist (Thurston *et al*, 2005; Bogdanovic *et al*, 2006; Augustin *et al*, 2009; Yuan *et al*, 2009; Thurston & Daly, 2012). Their monomeric structures are highly similar, but Ang1 predominantly exists in higher-order multimeric forms, while Ang2 mainly exists in dimeric form (Davis *et al*, 2003; Cho *et al*, 2004; Kim *et al*, 2005). Minimally tetrameric oligomerization of Ang1 was thought to be a requirement of its activity as a Tie2 agonist, while monomeric and dimeric Ang1 antagonized Tie2 activity in Ang2-like fashion (Davis *et al*, 2003).

For therapeutic use, recombinant variants or mimetics of multimeric Ang1 have been engineered, including Ang1* and Ang-F1-Fc-F1/Bow-Ang1 (Davis *et al*, 2003; Daly *et al*, 2013), MAT-Ang1 and COMP-Ang1 (Cho *et al*, 2004; Koh, 2013). Unlike these Ang1 variants, Vasculotide (VT) does not adopt the globular, 215-amino-acid-long, Tie2-binding ‘fibrinogen-like domains’ of native Ang1 (Davis *et al*, 2003; Cho *et al*, 2004). The development of VT was inspired by a 2004 paper, where several heptapeptides—with no sequence homology to Ang1—were identified to have Tie2-binding potential through an ELISA screen of a phage-displayed peptide library (Tournaire *et al*, 2004). Among them was ‘T7’ (HHHRHSF), which in its synthetic free form did not inhibit Ang1 or Ang2 binding to Tie2 in competition assays (Tournaire *et al*, 2004). By conjugating together four copies of ‘T7’, using an avidin backbone in the first-generation design, VT was developed with the aim of tetramerically binding and clustering Tie2 receptors in an ‘Ang1-like’ manner to activate Tie2 signaling (Van Slyke *et al*, 2009). The current generation of VT is PEGylated (Supplementary Fig S1): It employs a 4-armed maleimide-functionalized polyethylene glycol (PEG) backbone to link together four cysteine-capped T7 peptides, that is, CHHHRHSF (‘T7c’). This PEGylated VT reportedly activates Tie2 phosphorylation and induces Ang1-like cellular and physiological responses. For instance, in endotoxemic mice, VT prevented inflammatory induction of lung vascular hyperpermeability by preserving VE-cadherin-mediated EC junctions (David *et al*, 2011). In an *in vivo* model of abdominal sepsis, VT reduced intraperitoneal leukocyte influx through suppression of pro-inflammatory cytokines (e.g., TNF-α, IL-6) and endothelial adhesion molecules (e.g., ICAM-1 and VCAM-1) (Kumpers *et al*, 2011). In a mouse model of skin toxicity from cancer radiotherapy, VT promoted wound healing by reducing irradiation-induced inflammation (e.g., neutrophil recruitment) and microvascular damage (Korpela *et al*, 2014). The objective of our study was to harness these potentially promising vascular-stabilizing and anti-inflammatory properties of VT in developing an Ang1-mimetic therapeutic strategy for inhibiting early stages of metastatic spread.

## Results

### Vasculotide has permeability-limiting effects on endothelial cells and inhibits tumor cell extravasation *in vitro*

Vasculotide was previously shown to preserve endothelial barrier integrity when such cells are stimulated with sepsis-related mediators of vascular hyperpermeability, including thrombin (David *et al*, 2011). Thrombin is also a metastasis-associated factor that can promote endothelial adhesion and diapedesis of TCs (Nierodzik & Karpatkin, 2006).

**Figure 1:**
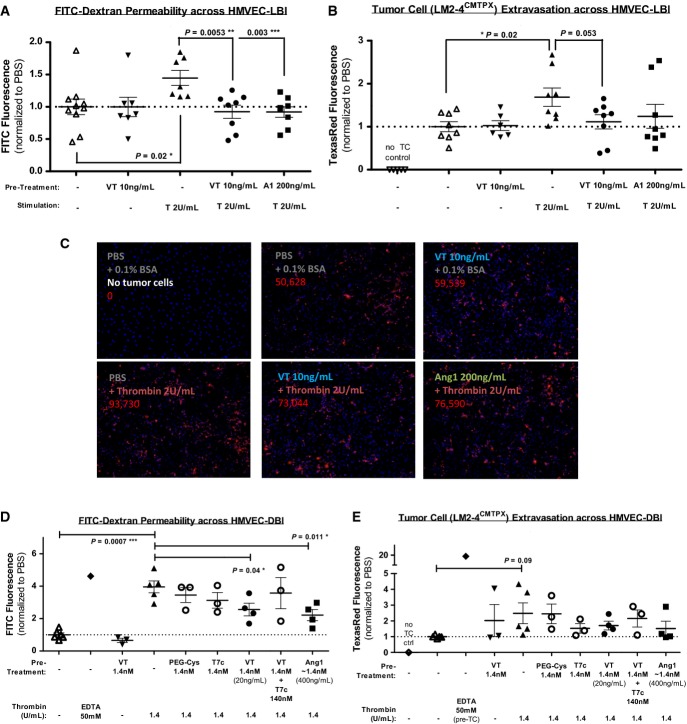
Vasculotide and Ang1 treatments counteract thrombin induction of trans-endothelium macromolecular permeability and tumor cell migration *in vitro*

Using *in vitro* modified Boyden chamber assays, where insert filter membranes were lined by confluent human microvascular ECs of either lung or dermal blood vessel origin (HMVEC-LBl, Fig1A–C; HMVEC-DBl, Fig1D and E), we observed that VT treatment was able to counteract thrombin-stimulated increases in transendothelial permeability of FITC-dextran (*P *< 0.05, Fig1A and D) and showed trends of reducing thrombin-stimulated migration of CMTPX-labeled TCs (Fig1B, C and E).

A–E Microvascular leak and tumor cell extravasation were modeled *in vitro* using modified Boyden chamber experiments where lung HMVECs (A–C) and dermal HMVECs (D–E) were grown to 100% confluence over 8-μm-pore insert membranes. ECs were first treated with Vasculotide (VT), PBS (vehicle/negative control), Ang1 (positive control), PEG-Cys (polyethylene glycol backbone), T7c (non-PEGylated CHHHRHSF peptides), or VT in the presence of 100-fold molar excess of T7c. The concentrations used, 10–20 ng/mL VT and 200–400 ng/mL Ang1, are estimated molar equivalents (0.71–1.43 nM). Thirty minutes later, ECs were stimulated with thrombin, 0.1% BSA (vehicle/negative control), or EDTA (positive control). Another 30mins later, the amount of FITC-dextran diffusion into the lower chambers (A, D) provides a measure of endothelial permeability. CMTPX-labeled tumor cells (TCs) were then dispensed into inserts, and the amount of TC fluorescence emitting from the underside of insert membranes after 20 h (B–C) or 28 h (E) reflects the efficiency of trans-endothelial TC migration. Representative fluorescent images (10×) of membrane undersides are shown in (C), where DAPI-stained nuclei of ECs/TCs are shown in blue, ‘extravasated’ CMTPX-labeled TCs are shown in red, and the # of CMTPX^+^ pixels are shown numerically in red. Means ± SEM are shown (A,B,D,E). Three experiments (twice with HMVEC-LBl and once with HMVEC-DBl) were run with *n* = 3–5 inserts (independent biological replicates) per group and analyzed by two-sampled unpaired *t*-tests.

Several structural components of VT were additionally tested as controls (Supplementary Fig S1): ‘T7c’ refers to non-PEGylated CHHHRHSF peptides, and ‘PEG-Cys’ refers to the PEG backbone alone. While these structural components individually contributed partial effects, the intact structure of VT appeared to be necessary for full anti-permeability effect (Fig1D). Moreover, saturation of VT's binding targets by 100-fold molar excess of T7c peptides dampened the effects of VT (Fig1D and E), suggesting that VT function is at least partially dependent on its T7 moieties.

### Breast cancer metastasis model: Vasculotide inhibits experimental LM2-4^luc^ metastasis to the lungs but not lymphatics

We next assessed the anti-metastatic potential of VT *in vivo* using three models of ‘experimental metastasis’. By injecting a fixed number of human breast (LM2-4^luc^), colon (HT29^luc^), or renal (SN12^luc^) cancer cells directly into the venous circulation of SCID mice, we modeled specifically the later steps of the ‘metastasis cascade’—TC extravasation and metastatic colonization—within specific host organs particularly susceptible to each cancer type.

We showed previously that sunitinib (SU) treatment prior to intravenous (IV) inoculation of LM2-4^luc^ cells, through pre-conditioning of the host environment, can lead to a promotion of experimental metastasis, especially in the lungs (Ebos *et al*, 2009)—results confirmed by others (Chung *et al*, 2012; Welti *et al*, 2012). Here, we tested whether concurrent VT therapy can be used to reverse this pro-metastatic potential of SU in the same model. LM2-4^luc^ is a metastatically aggressive luciferase-tagged derivative of the MDA-MB-231 cell line that was derived through serial *in vivo* selection of lung metastases (Munoz *et al*, 2006; Francia *et al*, 2011). While early passages of LM2-4^luc^ maintained a high propensity for lung colonization, later passages of LM2-4^luc^ reverted to its parental bias for lymphatic colonization (F.T. Wu, C.R. Lee and R.S. Kerbel, unpublished observations).

**Figure 2:**
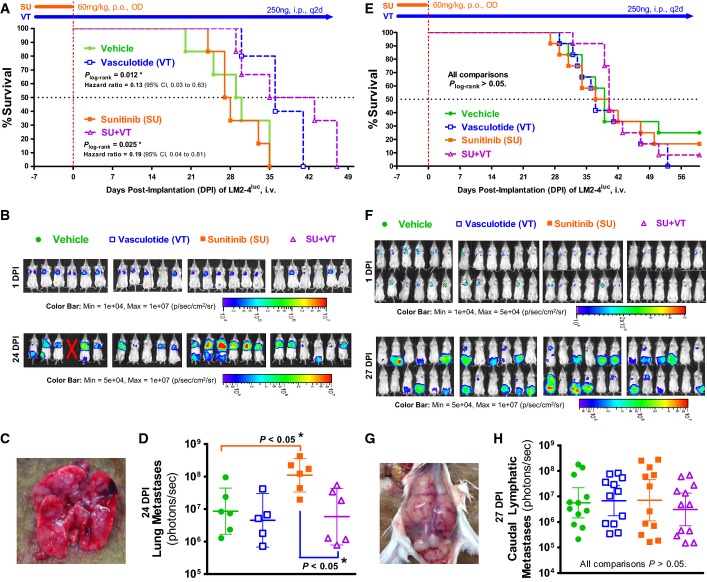
Vasculotide delays progression of experimental lung metastases but not lymphatic metastases from a human breast cancer cell line (LM2-4^luc^)

Figure2 panels A–D summarize an experiment where a predominance of lung-specific metastases developed after IV inoculation of early-passage LM2-4^luc^ cells into SCID mice, so that the main reason for sacrifice or mortality was labored breathing. In this case, long-term 250 ng/2d VT monotherapy conferred a significant survival benefit (*P *= 0.01), extending median survival by ∽20% (Fig2A). *In vivo* bioluminescent imaging (IVBI) recorded a trend of reduced lung metastases with VT monotherapy (Fig2B–D). As a concurrent therapy to SU (60 mg/kg/day) pretreatment, VT also prolonged median survival (Fig2A) by effectively suppressing SU-induced promotion of lung metastases, as seen by IVBI (*P *< 0.05, Fig2D). Interestingly, while SU pretreatment promoted lung metastases (Fig2D), it did not similarly promote lymphatic metastases (Fig2H).

A–H LM2-4^luc^ tumor cells were implanted intravenously into SCID mice, generating predominantly lung-specific metastases in one experiment (*n* = 5–6; A–D) and predominantly lymphatic-specific metastases in another experiment (*n* = 12; E–H). (C and G) Photographs of typical metastases in the lungs and caudal lymphatics, respectively. Kaplan–Meier survival analysis showed that Vasculotide (VT) significantly delayed mortality due to lung metastases (A) but not lymphatic metastases (E). Quantitative analysis of *in vivo* bioluminescent images taken at 24DPI (B) showed that concurrent VT treatment effectively reversed the sunitinib (SU)-induced acceleration of lung metastases (D). With regard to lymphatic metastases (F, H), SU treatment did not accelerate their progression. Geometric means ± 95%CI are depicted in (D and H); *P* values were derived by one-way ANOVA (D) and Kruskal–Wallis test (H). The same trends were reproduced in confirmatory experiments summarized in Supplementary Fig S5.

In another experiment (Fig2E–H), IV injection of later-passage LM2-4^luc^ cells into SCID mice led to extensive metastases in the lymphatics draining the tail vein, such that the main reason for sacrifice was hindlimb immobility. Here, of considerable interest, VT had no significant effect on survival rates (Fig2E) or the progression of lymphatic metastases (Fig2F–H)—thus implicating organ-specific or associated effects of VT on metastatic disease outcome.

### Colon cancer metastasis model: Vasculotide does not inhibit experimental HT29^luc^ metastasis to the liver or lymphatics

**Figure 3:**
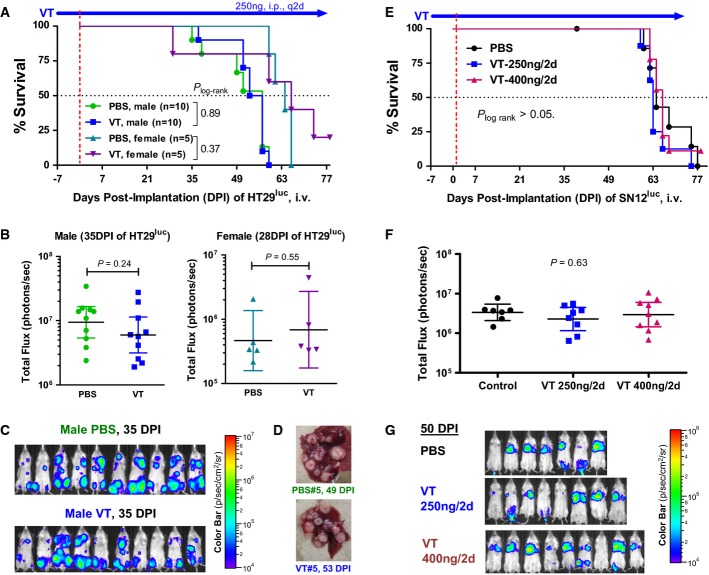
Vasculotide has no effect on experimental liver metastases from a colon cancer cell line (HT29^luc^), nor on experimental lung metastases from a renal cancer cell line (SN12^luc^)

Since VT was effective at inhibiting hematogenously disseminated lung metastases, we asked whether it could also inhibit hematogenously disseminated liver metastases. Four to five weeks after IV injection of human colon cancer HT29^luc^ cells (Hackl *et al*, 2013) into YFP-SCID mice, extensive experimental liver and lymphatic metastases could be observed (Fig3A–D). Sacrificial endpoints were defined by immobility or ≥ 20% weight loss. No significant differences between PBS (control) vs. VT-treated mice were seen in terms of survival rates (Fig3A), overall metastatic burden by whole-body IVBI (Fig3B and C), or liver-specific metastases by necropsy (Fig3D).

A–D An experiment where HT29^luc^ tumor cells were implanted intravenously into YFP-SCID mice. (A) Kaplan–Meier survival analysis. (B) Quantification of total metastatic burden in the liver and lymphatics, at 35 days post-implantation (DPI), from whole-body bioluminescent imaging (C). (D) Photograph of typical liver metastases.

E–G An experiment where SN12^luc^ tumor cells were implanted intravenously into YFP-SCID mice. (E) Kaplan–Meier survival analysis. (F) Quantification of total metastatic burden at 50DPI, mostly within the lungs, from whole-body bioluminescent imaging (G).

Data information: Geometric means ± 95%CI are depicted in B and F. *P* values were derived by *t*-tests (B) or one-way ANOVA (F).

### Renal cancer metastasis model: Vasculotide does not inhibit experimental SN12^luc^ metastasis to the lungs

A predominance of experimental lung metastases also developed after the tail-vein injection of human renal cancer SN12-PM6-L1^luc^ cells, referred to as ‘SN12^luc^’ hereafter (Jedeszko *et al*, 2015). SN12^luc^-derived lung metastases did not respond to VT treatment, whether given at the standard 250 ng/2 days or a higher 400 ng/2 days dose (Fig3E–G), in contrast to the responsiveness of LM2-4^luc^-derived lung metastases to 250 ng/2 days VT treatment (Fig2A–D). Since the same pulmonary endothelium was targeted in both cases, the variable *in vivo* efficacy of VT in curtailing lung metastases appeared to be dependent on the originating cancer cells.

### Endothelial cell-activating cytokines produced by Tie2^−^ tumor cells may influence the efficacy of Vasculotide *in vitro*

One way by which the originating TCs could influence the *in vivo* efficacy of VT might be through their differential production of EC-activating cytokines. To investigate this possibility *in vitro*, we performed modified Boyden chamber assays where we stimulated ECs with tumor cell-conditioned media (TC-CM)—that is, supplement-reduced EC growth media containing all the cytokines naturally secreted by LM2-4^luc^, SN12^luc^, or HT29^luc^ cells over 30 hours of hypoxic incubation (1% O_2_).

**Figure 4:**
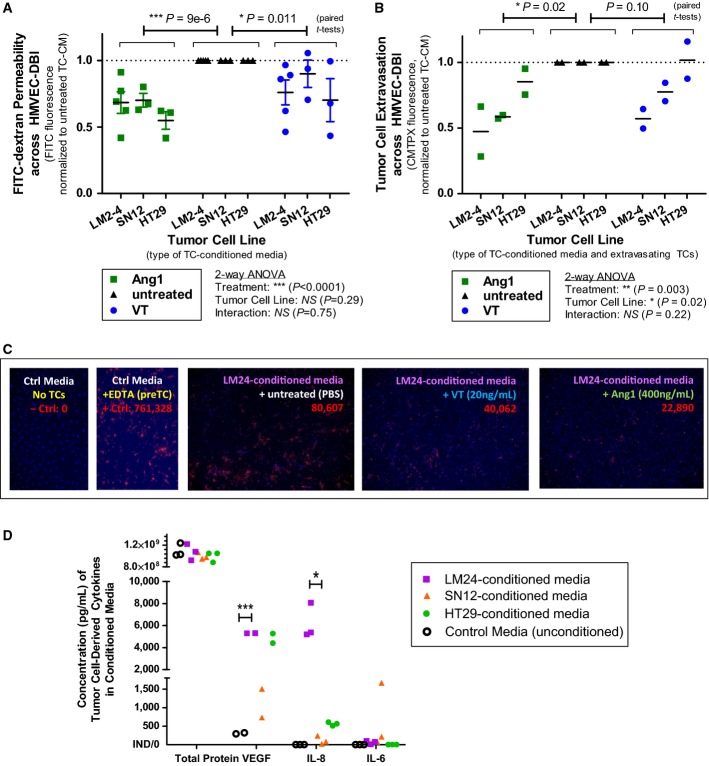
Endothelial cell-activating cytokines produced by Tie2^−^ tumor cells may influence the anti-permeability efficacy of Vasculotide and Ang1

In the presence of TC-CM stimulation, Ang1 or VT treatments reduced transendothelial macromolecular permeability (*P *< 0.05; Fig4A) and TC migration (*P *< 0.05 for Ang1 and *P *= 0.10 for VT; Fig4B and C). Interestingly, the degree of Ang1 or VT treatment efficacy varied depending on the type of TC-CM used (Fig4B); for instance, VT treatment achieved greater inhibition of TC migration, on average, in the presence of LM2-4^luc^-conditioned media (−43%) compared to SN12^luc^-conditioned media (−23%). We asked whether this could be due to differences in cytokine composition between the three TC-CM types.

A, B In modified Boyden chamber experiments, confluent dermal HMVECs were stimulated with tumor cell-conditioned media (TC-CM)—that is, media conditioned by either LM2-4^luc^, SN12^luc^, or HT29^luc^ tumor cells—and treated with 20 ng/mL Vasculotide vs. 400 ng/mL Ang1 (positive control) vs. PBS (negative control). Meta-analyses of treatment effects on dextran permeability (A) and tumor cell migration (B), where each datapoint represents the mean of an independent experiment, averaged over 2–3 biological replicates (inserts) per treatment group and normalized internally within that same experiment. Statistical significance of overall Ang1 or VT treatment effects was determined by paired *t*-tests, *n* = 11 (A) or 6 (B) experimental replicates.

C Representative fluorescent images (10×) of ‘extravasated’ CMTPX-labeled TCs (red) fixed on membrane undersides.

D LM2-4^luc^ produced the highest levels of VEGF and IL-8, but lower levels of IL-6, compared to other TC types; ***P < 0.001 and *P < 0.05 (unpaired *t*-tests).

By ELISA or flow cytometric bead-based immunoassays, we quantified the media concentrations of three permeability-inducing and/or pro-inflammatory cytokines, whose elevated levels in cancer patients often correlate with disease progression and poor prognosis: VEGF, IL-8, and IL-6 (Ferrara, 2005; Kut *et al*, 2007; Lippitz, 2013). Both VEGF and IL-8 can directly activate ECs, via VEGFR2 and CXCR1/CXCR2 receptors, respectively (Kim *et al*, 2001a; Le Guelte *et al*, 2011). In contrast, IL-6 primarily targets IL-6R^+^ leukocytes/TCs and only indirectly affects IL-6R^−^ ECs (Romano *et al*, 1997; Lo *et al*, 2011). We found that the media concentrations of VEGF and IL-8, but not IL-6, were considerably higher in LM2-4-conditioned media compared to other TC-CM types (Fig4D)—suggesting that the contribution of LM2-4^luc^-derived VEGF/IL-8 to endothelial gap formation (Fig4A) and TC diapedesis (Fig4B) may be particularly amenable to counteraction by Ang1 or VT.

### Spontaneous breast cancer metastasis model: Vasculotide treatment does not suppress orthotopic primary tumor growth but inhibits metastasis to the lungs

Orthotopic implantation experiments were also performed with the LM2-4^luc^ cells, to further explore the effects of VT on primary tumor growth vs. spontaneous metastatic dissemination.

**Figure 5:**
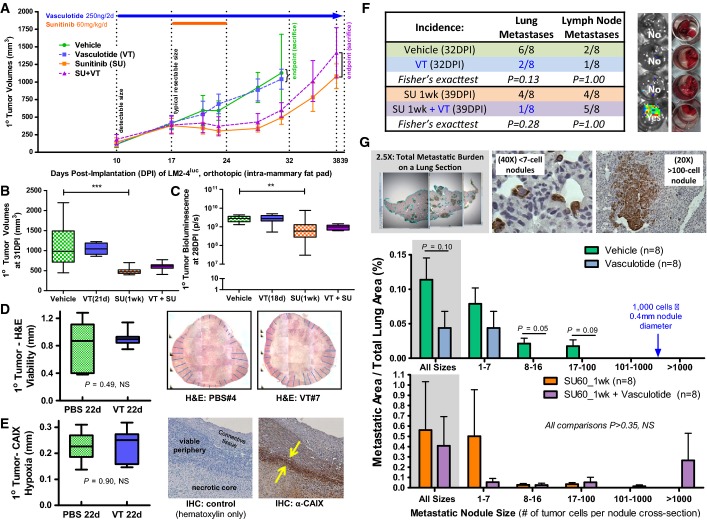
Vasculotide delays the dissemination of spontaneous lung metastases from orthotopic breast cancer (LM2-4^luc^) xenografts without having effect on primary tumors

The first experiment involved VT and SU treatments in the presence of an unresected primary tumor (Fig5). At 10 days post-implantation (DPI), when primary tumors reached an average size of ∽175 mm^3^, mice were randomized into groups and chronic VT therapy was initiated. At 17DPI, when tumors reached an average size (∽400 mm^3^) where tumor debulking is typically required for long-term metastasis experiments, a 7-day anti-angiogenic SU treatment was initiated in lieu of surgical resection. SU effectively stabilized primary tumor growth (Fig5A–C). In contrast, VT did not alter the growth kinetics of primary tumors, as measured by volume or bioluminescence (Fig5A–C). This was consistent with *in vitro* observations from an MTS cell viability assay (Supplementary Fig S2), where VT had no direct cytotoxic effect on Tie2^−^ TCs and did not interfere with SU inhibition of Tie2^+^ ECs. Histological analysis of primary tumors showed that the extent of tumor cell viability (assessed by H&E staining; Fig5D) and tumor hypoxia (assessed by IHC staining of CAIX, a target of the hypoxia-inducible transcription factor, HIF-1; Fig5E) also remained unchanged after 22 days of VT treatment.

A–C Vasculotide (VT) did not alter the growth kinetics of orthotopic LM2-4^luc^ tumors; *n* = 8 mice per group. Sunitinib (SU) treatment significantly lowered primary tumor burden as measured by volume (B: one-way ANOVA, *P* < 0.01) or bioluminescent activity (C: one-way ANOVA, *P* < 0.001).

D VT treatment did not alter the mean thickness of the viable peripheral regions of primary tumors, as quantified by H&E histological staining.

E VT treatment did not alter the mean thickness of the CAIX^+^ hypoxic edge between the viable periphery and necrotic core of a primary tumor; IHC control and sample images taken at 5× are shown.

F *Ex vivo* bioluminescent imaging suggests that VT treatment lowered the proportion of mice with metastatic involvement in the lungs, without the corresponding effect on lymphatic metastases. Representative bioluminescent vs. photographic images of dissected lungs in a 24-well plate are shown.

G Human LM2-4^luc^ cells can be identified on histological sections of mouse lung tissue by IHC staining of human vimentin, a cytoskeletal protein. Size distribution analysis show VT monotherapy reducing the occurence of bigger metastatic nodules.

Data information: Means ± SD are depicted in (A). Means ± SEM and *P* values from *t-*tests are depicted in (B–E and G).

However, as detected by organ-specific *ex vivo* bioluminescent imaging (EVBI) upon sacrifice, there were trends of reduced incidences of metastatic involvement in the lungs, but no change in the lymph nodes, of the VT-treated groups, compared to their vehicle or SU monotherapy counterparts (Fig5F). IHC-based size distribution analysis of metastatic nodules suggests that VT monotherapy delayed the seeding or progression of lung micrometastases, with larger nodules apparent only in the vehicle group and not the VT monotherapy group (*P *= 0.05–0.09; Fig5G). Although a trend was observed by global EVBI analysis (Fig5F), the anti-metastatic benefit of concurrent VT treatment was not detectable by regional IHC analysis (Fig5G).

### Combining Vasculotide with adjuvant anti-angiogenic therapy to target residual and micrometastatic disease after surgical resection of primary breast tumors

**Figure 6:**
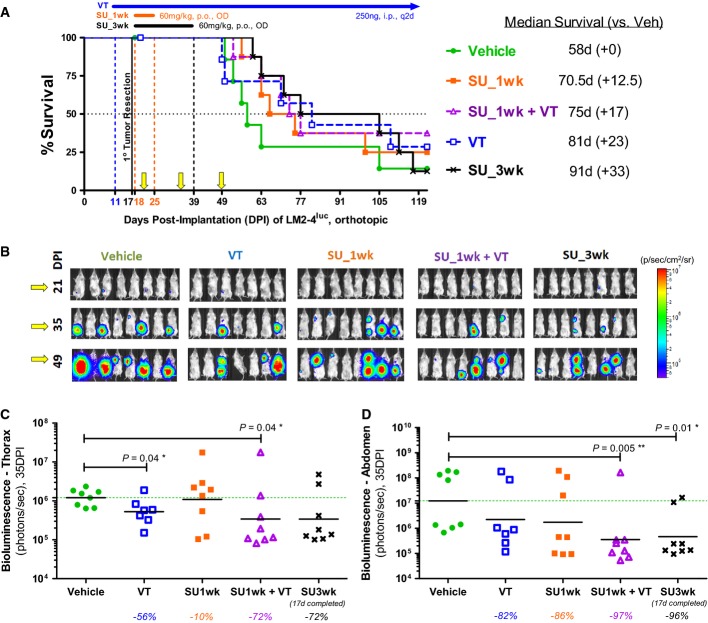
Combining perioperative Vasculotide treatment with adjuvant sunitinib treatment after surgical resection of LM2-4^luc^ primary tumors

A second orthotopic implantation experiment involved surgical resection of primary LM2-4^luc^ tumors (∽400 mm^3^) at 17DPI (Fig6) to model post-operative metastases arising from minimal residual disease (MRD). MRD includes the regrowth of incompletely resected mammary tumors, as well as circulating or disseminated tumor cells released from primary tumors prior to or at the time of surgical resection. VT was initiated 6 days prior to surgery and maintained chronically, while adjuvant SU (1-week or 3-week 60 mg/kg/day) treatments were initiated 1 day post-surgery (Fig6A).

A Kaplan–Meier survival curves. Number of days gained in median survival due to treatments is listed in brackets. Yellow arrows mark the timepoints at which the bioluminescent images were taken.

B Bioluminescent images of tumor burden.

C–D Total bioluminescent fluxes, at 35 days post-implantation (while the ‘SU_3 wk’ group was still receiving 60 mg/kg/day sunitinib treatment), were separately quantified from the thoracic (C) and abdominal (D) regions. Geometric means and *P* values from Mann–Whitney tests are depicted in (C and D). Percentages listed below the x-axes in panels (C and D) refer to treatment-associated changes in the geometric mean bioluminescence, relative to the vehicle group.

Two kinds of post-operative tumor burden, temporally and spatially distinct, were evident by bioluminescent imaging (Fig6B): (i) aggressive abdominal tumor burden—consisting of right inguinal mammary tumor regrowths, as well as metastatic expansions of pre-surgically invaded regional lymph nodes; and (ii) late-developing thoracic tumor burden—comprising lung metastases and axillary/brachial lymph node involvement. Within the critical 2.5 weeks post-surgery, during which we will contrast the effects of VT and SU treatment, locoregional proliferation/growth was the dominant driving force behind fast-expanding abdominal tumors, while in the thorax, TCs were still in the early stages of dissemination (extravasation, seeding and early colonization, etc.).

First, we observed that adjuvant SU treatment was more effective at inhibiting abdominal tumor growth (Fig6D) than thoracic metastatic dissemination (Fig6C). We also noted the brevity of SU treatment efficacy—abdominal tumors in the ‘SU_3 wk’ group appeared to be stabilized so long as SU treatment was maintained (Fig6B at 35DPI), but aggressively rebounded after cessation of SU treatment (Fig6B at 49DPI). This transiency in tumor growth suppression by SU accounts for the longer median survivals associated with longer treatment duration (Fig6A): 1-wk and 3-wk SU treatments extended median survival by 12.5 days (*P*_Gehan-Breslow-Wilcoxon_ = 0.17) and 33 days (*P*_GBW_ = 0.08), respectively. At 10 days after discontinuation of 1-week SU therapy, its transient inhibitory effect on abdominal tumor growths has greatly diminished (Fig6D), while no benefit was observed with respect to thoracic metastases (Fig6C).

In contrast, long-term VT monotherapy was more effective at inhibiting thoracic metastatic dissemination (Fig6C, *P* = 0.04) than abdominal tumor growth (Fig6D, *P* > 0.05). The survival advantage associated with VT monotherapy was not statistically significant despite an extension in median survival of 23 days (Fig6A, *P*_log-rank_ = 0.42). However, concurrent VT therapy conferred additive benefits—combining VT with 1-wk SU therapy, it lowered tumor bioluminescence to a similar extent as 3-wk SU monotherapy (Fig6C and D).

### Vasculotide does not induce Tie2 phosphorylation *in vitro* or *in vivo*

**Figure 7:**
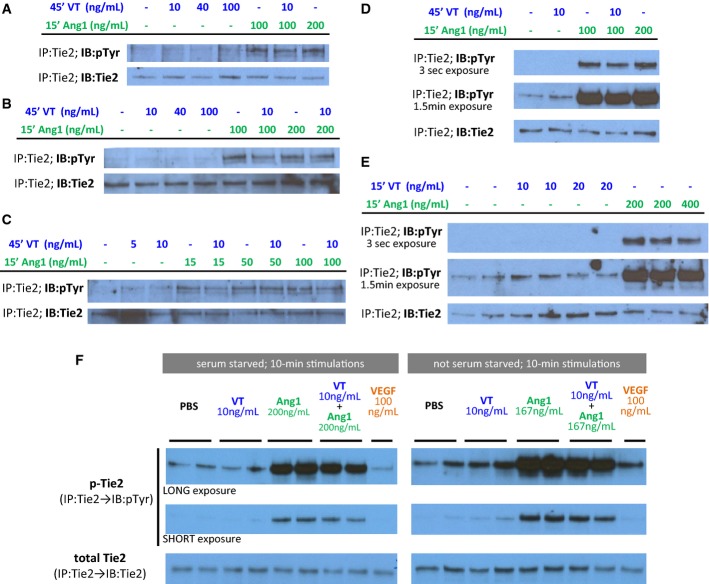
*In vitro* Vasculotide vs. Ang1 stimulations: effects on Tie2 receptor phosphorylation in primary endothelial cells—HUVECs (A-E) and HMVEC-DBls (F)

To follow-up on previous characterizations of VT as a Tie2-specific agonist, we stimulated primary human endothelial cells *in vitro* to directly assess the effects of VT on Tie2 phosphorylation status (Fig7). Ang1, as our positive control, strongly and consistently activated Tie2 phosphorylation across the concentrations tested (15–400 ng/mL or 0.05–1.43 nM). VEGF, which is not a ligand of Tie2, was used as a negative control. Unexpectedly, VT treatment did not lead to any appreciable induction of Tie2 phosphorylation under any conditions tested in this study—whether given at concentrations of 5, 10, 20, 40, or 100 ng/mL (0.36–7.1 nM); at different timepoints (10, 15, or 45 min); with or without serum and supplement starvation; in the absence or presence of concurrent Ang1 stimulation; and in ECs of venous (HUVEC) or microvascular (HMVEC-DBl) origin (Fig7). Importantly, even though VT (10–20 ng/mL) and Ang1 (200–400 ng/mL) both functionally inhibited endothelial permeability in Boyden chamber assays where HMVEC-DBl was used (Figs1 and 4), the same concentration of VT (10 ng/mL) did not have similar activity as equimolar Ang1 (200 ng/mL or 0.7 nM) at the upstream level of Tie2 phosphorylation in the same dermal microvascular ECs (Fig7F).

A–F Primary human umbilical vein endothelial cells (HUVECs) or primary human dermal microvascular endothelial cells (HMVEC-DBls) were stimulated at 80% confluency with Vasculotide (treatment), Ang1 (positive control; ligand of Tie2), PBS (vehicle control), or VEGF (negative control; non-ligand of Tie2). Tie2 was immunoprecipitated from cell lysates and immunoblotted for total Tie2 or phosphotyrosine. Vasculotide treatment did not lead to increases in the pTie2/Tie2 ratio in HUVECs after 45-min (A–D) or 15-min (E) treatments, with (E) or without (A–D) serum/supplement starvation. Vasculotide also did not activate Tie2 receptors in HMVEC-DBls, whether in the presence or absence of serum, growth factor, and cytokine supplements (F).

Source data are available online for this figure.

**Figure 8:**
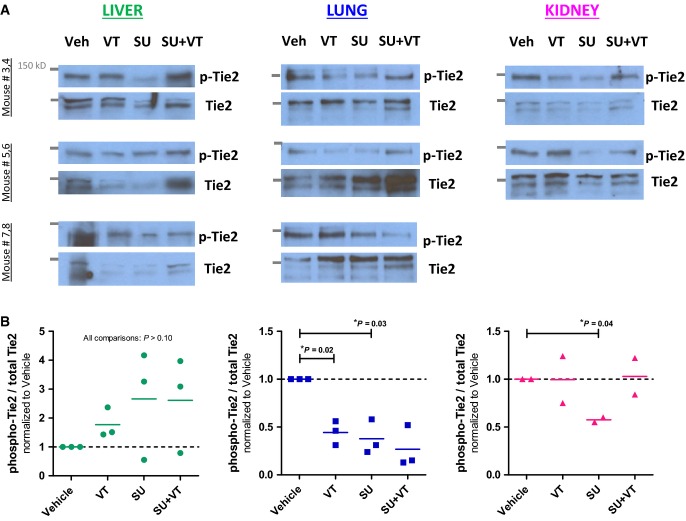
*In vivo* Vasculotide treatments: effects on Tie2 receptor phosphorylation in vascular-rich organs (liver, lungs, and kidneys)

Since VT did not appear to be a direct Tie2 agonist within a reductionistic *in vitro* system of cultured ECs (Fig7), we asked whether VT treatment may still indirectly lead to increased Tie2 phosphorylation within a physiological *in vivo* context. Tumor-free mice were treated for 1 week with either vehicle, VT (250 ng/2 days), SU (60 mg/kg/day), or VT+SU and then sacrificed 5 h after their last doses of treatment. Tissue homogenates derived from EC-rich organs (livers, lungs, kidneys) were subjected to Tie2 immunoprecipitation and then immunoblotted for phosphorylated Tie2 (Fig8: ‘p-Tie2’) or total Tie2 (Fig8: ‘Tie2’). Overall, *in vivo* VT treatment—whether as a monotherapy or when combined with SU—did not increase the ratio of phosphorylated Tie2 relative to total Tie2; in fact, lung-specific pTie2/Tie2 was significantly lower in the ‘VT’ group vs. ‘vehicle’ group (Fig8).

Healthy non-tumor-bearing mice (*n* = 8–9 per group) were treated for 1 week with vehicle (Veh); Vasculotide 250 ng/2 days IP (VT); sunitinib 60 mg/kg/day PO (SU); or VT + SU. Tie2 immunoprecipitates from lung, kidney, or liver homogenates were pooled from two mice per treatment group and immunoblotted with an anti-Tie2 antibody (‘*Tie2*’) or an anti-phosphotyrosine antibody (‘*p-Tie2*’).

Densitometry analysis of immunoblots from (A). pTie2/Tie2 ratios were internally normalized to the vehicle group within each blot. Means ± SEM and *P* values (unpaired *t*-tests) are depicted.

Source data are available online for this figure.

### Vasculotide does not bind the extracellular domain of Tie2

**Figure 9:**
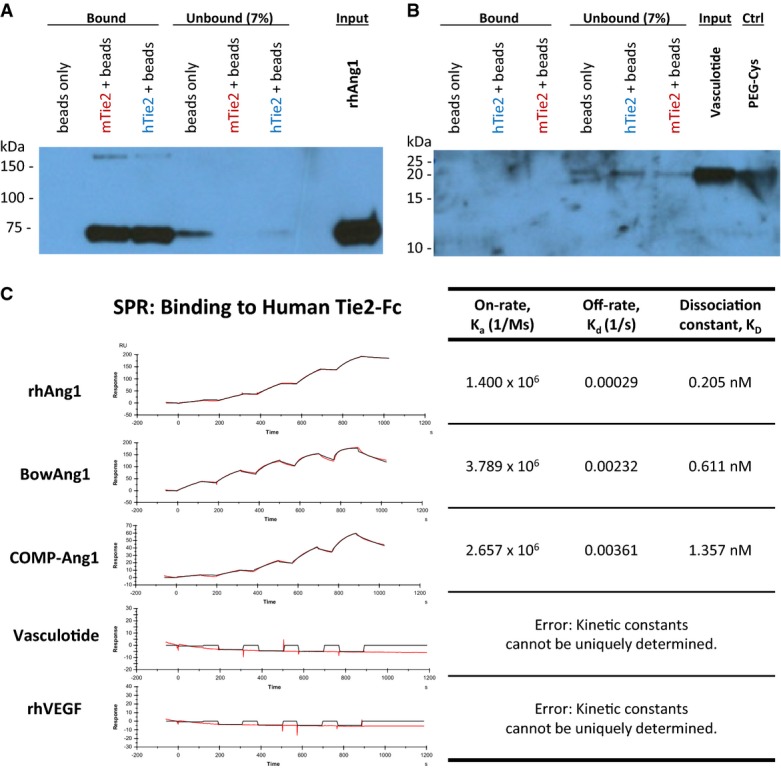
Binding of Vasculotide vs. Ang1 variants to the extracellular domain of Tie2

To better understand the difficulties we had in observing Tie2 activation by VT, we sought to characterize the binding affinity and kinetics of VT to Tie2. Purified human or mouse Tie2-Fc (i.e., Fc-conjugated extracellular domains of Tie2) were first coupled to protein A sepharose beads for pull-down experiments (Fig9A and B) or immobilized on a Biacore CM5 sensor chip for surface plasmon resonance (SPR) experiments (Fig9C and Supplementary Fig S3). In the pull-down assays, human and mouse Tie2-Fc effectively pulled down the majority of available recombinant human Ang1 (rhAng1), but did not pull down any appreciable levels of VT (Fig9A and B). Likewise, SPR experiments—performed at physiological conditions of 37°C and pH 7.4—showed rhAng1, BowAng1, and COMP-Ang1 to be high-affinity binders (dissociation constants, K_D_, of 0.2–1.4 nM) to human and mouse Tie2-Fc (Fig9C and Supplementary Fig S3B), but no binding to Tie2-Fc was detectable for VT, VEGF, PEG-Cys, or T7c (Fig9C and Supplementary Fig S3).

A–B Pull-down assay. Input ligands—rhAng1 (A) and VT (B)—were incubated with either protein A sepharose beads only, bead-conjugated mouse Tie2-Fc (mTie2), or bead-conjugated human Tie2-Fc (hTie2). The ‘bound’ fraction shows the total amount pulled down by beads. The ‘unbound’ fraction is 7% of residual/free ligands remaining in the supernatant.

C Quantitative kinetics analysis by SPR. All analytes were run at increasing concentrations of 0.625, 1.25, 2.5, 5, and 10 nM, at 37°C and pH 7.4. The positive controls—rhAng1, BowAng1, COMP-Ang1—all bound strongly to immobilized hTie2-Fc. There was no detectable binding of VT, or the negative control, rhVEGF, to immobilized hTie2-Fc.

Source data are available online for this figure.

## Discussion

We report here five new findings relevant to therapeutic manipulation of the vasculature for cancer treatment, especially of metastatic disease. First, VT inhibits vascular permeability to the extravasation of tumor cells *in vitro*. Second, consistent with the aforementioned findings, VT inhibits the early stages of the metastatic process *in vivo* and thus may be potentially effective as an adjuvant therapy; in contrast, it does not inhibit tumor growth *per se*, for example, of established primary tumors. Third, the anti-metastatic effects of VT appear to be organ and tumor dependent, for example, inhibition of lung metastasis of breast cancer was observed but not of liver metastasis by colorectal cancer cells nor of lung metastasis by renal cell carcinoma cells. Fourth, these aforementioned effects appear to be independent of binding to or activation of Tie2 and thus may work through a different and thus potentially novel mechanism. Fifth, a potentially important ‘off-target’ effect of a commonly used anti-angiogenic TKI, sunitinib, was observed—inhibition of Tie2 phosphorylation.

This study addresses a gap in cancer therapy research, where efforts have largely focused on identifying drugs that limit the local malignant growth of tumors, resulting in a paucity of treatments that limit the distant metastatic spreading of cancer (Steeg, 2012). For instance, sunitinib (SU), an anti-angiogenic TKI that target the VEGF receptors (VEGFRs), repeatedly failed in multiple phase III clinical trials and preclinical models of advanced metastatic breast cancer, whereas, in contrast, it demonstrated potency in inhibiting angiogenesis-dependent growth of primary breast tumors in preclinical models (Abrams *et al*, 2003; Guerin *et al*, 2013). Recent preclinical studies suggest that many anti-angiogenic TKIs may inadvertently destabilize the normal microvasculature of distant host organs to facilitate TC extravasation and promote paradoxical pro-metastatic side effects, for example, by targeting pericytes (Ebos *et al*, 2009; Chung *et al*, 2012; Cooke *et al*, 2012; Welti *et al*, 2012).

With this report, we present a novel anti-metastatic therapy, where we seek to curtail metastatic *dissemination*—in particular, TC extravasation through the host microvasculature of distant organs—rather than the *growth per se* of secondary tumors.

Vasculotide (VT) was purported to be an Ang1-mimetic Tie2-specific agonist (David *et al*, 2011; Kumpers *et al*, 2011; Korpela *et al*, 2014). We found that VT was able to impair TC diapedesis *in vitro* by way of improving endothelial barrier integrity. *In vivo*, VT subdued the pro-metastatic effects of SU treatment, without interfering with the anti-angiogenic growth-inhibitory effects of SU on primary tumors, in mouse models of breast cancer metastasis using the LM2-4^luc^ human cancer cell line. However, an in-depth investigation into the putative mechanism of action of VT did not yield any evidence of direct binding to Tie2 or agonistic induction of Tie2 phosphorylation. We conclude that the vascular-stabilizing effects of VT do not occur through Tie2 agonism; alternative mechanism(s) of action have yet to be determined.

Meanwhile, there appeared to be organ-specific heterogeneity in the anti-metastatic efficacy of VT *in vivo*, possibly due to biological and structural differences in the type of endothelium being targeted. We observed that VT most effectively inhibited LM2-4^luc^ extravasation to the lung, but was ineffective against HT29^luc^ (a human colon cancer cell line) extravasation to the liver or LM2-4^luc^/HT29^luc^ infiltration of lymph nodes in mice. Moreover, not all cancer models with metastatic tropism to the lung uniformly benefited from VT treatment. In our study, lung metastases derived from LM2-4^luc^ (triple-negative breast cancer cells that secrete high levels of VEGF and IL-8), but not SN12^luc^ (*VHL*-wild-type renal cancer cells that secrete low levels of VEGF and IL-8), responded to VT treatment.

### Vasculotide inhibits metastatic dissemination by targeting the established host vasculature

Using modified Boyden chamber experiments, we showed as a proof-of-concept that VT treatment could limit TC diapedesis across dermal or lung HMVECs that had been activated by various cancer-associated cytokines including thrombin, VEGF, and IL-8. The efficacy of VT in our *in vitro* dextran permeability assays can be ascribed to the ability of VT to prevent inter-EC gap formation (David *et al*, 2011). The efficacy of VT in our *in vitro* TC migration assays may additionally reflect an attenuation of EC surface adhesion molecules that can facilitate TC diapedesis—for example, VCAM-1 and ICAM-1 (Kumpers *et al*, 2011).

Given the lack of inhibitory effects by VT on the viability of proliferating ECs and TCs *in vitro*, it was not surprising that VT did not inhibit primary mammary tumor growth rates *in vivo*. Accordingly, the inhibition of secondary tumors in the lungs by VT also would not have occurred through anti-angiogenic targeting of sprouting ECs or cytotoxic targeting of proliferating TCs. Instead, we propose that VT delayed TC extravasation or metastatic seeding in the lungs *in vivo* mainly by stabilizing the barrier function of mature blood vessels in the lungs (e.g., by limiting permeability). The type of vascular endothelium traversed by TCs en route to their metastatic destination (Supplementary Fig S6) is likely to influence the efficacy of VT (Strell & Entschladen, 2008). The continuous non-fenestrated blood vessels of the pulmonary capillary beds were found to be most ‘targetable’ by VT. In comparison, VT treatment did not discernibly affect TC transit across the discontinuous fenestrated endothelium of the hepatic sinusoids or the loose cell–cell junctions of lymphatic capillaries.

Additionally, TC-derived permeability-inducing cytokines may also affect EC barrier-enhancing potential of VT treatment. *In vitro*, VT inhibition of TC migration across dermal HMVECs was greater in LM2-4^luc^-conditioned media containing high levels of VEGF/IL-8 than in SN12^luc^-conditioned media containing low levels of VEGF/IL-8. *In vivo*, VT effectively inhibited the progression of lung metastasis from LM2-4^luc^ cells but not from *VHL*-wild-type SN12^luc^ cells. An interesting possibility is that VEGF/IL-8-driven TC extravasation across lung HMVECs may be particularly amenable to VT counteraction. While VEGF and IL-8 are two of the most potent and well-studied inducers of vascular permeability, there are potentially other factors that could also significantly modulate the efficacy of VT.

### Is Vasculotide a Tie2 agonist or an Ang1 mimetic?

By definition, a Tie2 agonist is a ligand that (a) directly binds to Tie2 receptors and (b) activates the tyrosine kinase activity and autophosphorylation of Tie2. In the assays performed for this study, we found no evidence of VT fulfilling either of these necessary requirements.

To our knowledge, the direct binding of the full VT to Tie2 has not been formally described or quantified. In their first paper, Van Slyke *et al* observed a faint Western blot signal when 20 nM of single-armed VT (i.e., one T7c peptide conjugated to a linear maleimide–PEG–biotin) was used to pull-down Tie2 from whole-cell lysates of EA.hy926 cells (Van Slyke *et al*, 2009). In contrast, the direct binding of tetravalent VT—whether avidin-conjugated (Van Slyke *et al*, 2009) or PEG-conjugated (David *et al*, 2011; Kumpers *et al*, 2011; Korpela *et al*, 2014)—to purified Tie2 has not been experimentally confirmed to our knowledge. The two direct binding assays used in this study, namely a pull-down assay and SPR analysis using purified Tie2-Fc, both revealed no detectable or quantifiable binding of PEGylated VT to the extracellular domain of Tie2.

Also unexpected was the undetectable binding of T7c peptides to Tie2-Fc by SPR. The main focus of the original paper that discovered T7 (Tournaire *et al*, 2004) was actually on a different peptide called ‘T4’ (NLLMAAS) and its potential use as a Tie2 inhibitor/antagonist. When they initially screened phage-displayed heptapeptides by ELISA for Tie2-binding potential, T4 and T7 were among four candidates selected for further study. These candidate peptides, in their synthetic free form, were then subjected to competition assays by ELISA and SPR to show that T4 was able to competitively inhibit Ang1 or Ang2 binding to Tie2 (*K*_*i*_ ∽ 0.3 mM), while T7 was not (tested up to 1 mM). In essence, these competition assays confirmed Tie2 binding for T4, but not for T7.

Our repeated investigations into the induction of Tie2 phosphorylation *in vitro* (analyzing primary venous and microvascular ECs treated directly with VT) and *in vivo* (analyzing lung, kidney, and liver tissue from mice treated with VT) also did not confirm the anticipated agonistic activity of PEGylated VT on Tie2. Of the three published papers on PEGylated VT, only the most recent study showed direct testing of VT on ECs *in vitro*, albeit on hTERT-immortalized ECs (Korpela *et al*, 2014). To show Tie2 phosphorylation or dependency, the older studies relied on *in vivo* systems, where indirect dependency and sample heterogeneity (e.g., variability in EC content) are inherently more difficult to exclude or account for, particularly when crucial controls are absent.

Altogether, Tie2 agonism is unlikely to be the mechanism of VT's vascular-stabilizing effects. Since many other signaling pathways (e.g., VEGF, thrombin, IL-8, Ca^2+^) crosstalk and converge to govern the same biological responses that are regulated by Ang-Tie2 signaling (Le Guelte *et al*, 2011; Koh, 2013), it would be premature to categorize VT as an Ang1 mimetic based on similarity in downstream function alone.

Further studies will be needed to determine whether VT has any other molecular binding partners, and if so, the binding affinity, kinetics, specificity, and biological significance of such interactions. Studies are in progress to elucidate possible mechanisms by which VT can cause the functional effects we have observed.

### Does sunitinib inhibit tumor growth, by targeting the developing tumor neovasculature, while potentially exacerbating tumor spread?

Many anti-angiogenic TKI therapies have underperformed clinically for patients with advanced metastatic breast cancer (MBC)—especially with sunitinib (SU) treatment, which when given as a monotherapy or combined with conventional chemotherapies has repeatedly failed in multiple phase III clinical trials (Ebos & Kerbel, 2011; Mackey *et al*, 2012). These clinical results were recently replicated in a preclinical model of postsurgical advanced MBC (Guerin *et al*, 2013). As discussed previously (Guerin *et al*, 2013), primary and secondary tumors may have divergent responses to anti-angiogenic therapies due to differences in their relative dependency on angiogenesis.

Another possible explanation for the apparent insensitivity of MBC to TKI treatments is that the growth of secondary tumors post-metastatic colonization of the lung/liver may still be susceptible to the intended anti-angiogenic inhibition, but that these growth-inhibitory benefits may be diluted by concurrent unintended drug effects that promote tumor spread. This appears to be the case in our resected orthotopic LM2-4^luc^ experiment, where adjuvant SU treatment transiently suppressed the locoregional growth of residual abdominal tumors, but was ineffective against the distal dissemination of thoracic tumors. Counterproductive dissemination-promoting side effects may explain the clinical observations from a phase II study of advanced MBC where response rates to sunitinib differed between patients with locoregionally growing superficial metastatic disease (20%) and patients with distally disseminated visceral metastatic disease (9%) (Yardley *et al*, 2012).

An interesting and unexpected finding from our study is that Tie2 dephosphorylation in the host vasculature may be yet another unintended consequence of TKI treatments which could promote metastatic seeding. This adds to other mechanisms previously proposed: therapy-induced tumor hypoxia could activate HIF-1 and HGF/Met pathways that increase tumor invasiveness and TC intravasation (Paez-Ribes *et al*, 2009; Cooke *et al*, 2012); host responses that upregulate circulating pro-angiogenic factors (Ebos *et al*, 2007) could enhance vascular permeability and TC diapedesis; and the destabilization of inter-EC junctions and pericytes in host organs could promote TC arrest or extravasation (Chung *et al*, 2012; Welti *et al*, 2012).

Also, organ specificity in relation to the pro-metastatic potential of SU was noted in this study—SU pretreatment preferentially accelerated experimental lung metastasis, but not lymphatic metastasis, from IV-injected breast cancer cells. This finding adds to a prior report where SU treatment of pancreatic neuroendocrine tumors increased metastasis to the liver but not the lymph nodes (LNs) (Paez-Ribes *et al*, 2009). There are at least two plausible explanations for this differential impact of SU on hematogenous metastasis (to lung/liver) vs. lymphogenous metastasis (to LNs). Paez-Ribes *et al* (2009) hypothesized that the concomitant disruption of lymphatic EC signaling via VEGFR3 inhibition by SU, a broad-spectrum TKI, may have prevented its general pro-metastatic potential from actualizing through the lymphogenous route. Moreover, PDGFR inhibition and pericyte depletion by SU had been implicated in its augmentation of lung metastasis (Cooke *et al*, 2012; Welti *et al*, 2012); since lymphatic vessels are not covered by pericytes, one would not expect a similar enhancement of LN metastasis through this mechanism.

### Complementary anti-metastatic strategies: combining host-targeting vascular stabilization with tumor-targeting anti-angiogenic therapy

In summary, our results suggest that the mechanisms of action and therapeutic effects of sunitinib (SU) treatment were distinct from—and perhaps complementary to—that of VT treatment. Anti-angiogenic SU therapy inhibits tumor growth, at least in part by targeting the tumor neovasculature to suppress the ‘angiogenic switch’ (Bagri *et al*, 2010), but may inadvertently promote metastatic dissemination. EC-stabilizing VT therapy targets the normal vasculature of host organs, especially in the lungs, to prevent metastatic extravasation, while having no effect on tumor growth per se. As such, a potentially promising application for anti-metastatic VT therapy to consider would be in the post-surgical micrometastatic disease setting, where VT could reduce the potential disadvantages associated with discontinuous adjuvant anti-angiogenic sunitinib therapy, or surgery-induced spreading of residual tumor cells (Goldfarb & Ben-Eliyahu, 2006).

## Materials and Methods

### *In vitro* modified Boyden chamber experiments

Endothelial cells (ECs) were grown on cell culture inserts with uncoated 8-μm-pore PET filter membranes to 100% confluency. See Supplementary Fig S4 for detailed timelines. Experiments began with a media change, introducing EC-stabilizing treatments (VT (Bachem); or rhAng1 (R&D Systems)) and EC-destabilizing stimulants (thrombin; EDTA; or tumor cell (TC)-conditioned media) into both upper and lower chambers. Permeability assays: A total of 100 μg of FITC-dextran (Sigma FD-20S) was dispensed into the upper chamber media. After 30 min of thrombin stimulation or 4–6 h of TC-CM stimulation, FITC fluorescence (ex/em = 490/520 nm) was measured from a 50 μl sample of the lower chamber media. Tumor cell extravasation assays: A total of 4 × 10^4^ TCs freshly labeled with CellTracker™ Red CMPTX (Invitrogen C34552: ex/em = 577/602 nm) were seeded into each insert. Where EDTA was used in the permeability assay, the assay media was changed to remove EDTA before introducing TCs. After a 15- to 30-h incubation, the ECs and non-migrated TCs above insert membranes were removed with a cotton swab, whereas migrated/‘extravasated’ TCs and ECs below insert membranes were fixed in 4% PFA. Fixed membranes were mounted onto slides with DAPI stain. CMPTX fluorescence from migrated TCs was then quantified from 10× microscopy images using a MATLAB (MathWorks, Natick, MA, USA) script; five images (technical replicates) were analyzed per insert.

### Animal experiments

*In vivo* experiments were performed in strict accordance with protocols approved by the Sunnybrook Research Institute Animal Care Committee, accredited by the Canadian Council of Animal Care. All surgical, imaging, and euthanasia procedures were performed under inhaled isoflurane anesthesia. Experimental metastasis models involved injections of 10^6^ tumor cells, suspended in 200 μl serum-free DMEM, into the mouse tail vein. LM2-4^luc^ implantations were performed on 6 to 8-week-old female CB-17 SCID mice (Charles River Canada). HT29^luc^ implantations were performed on 19- to 20-week-old male and 11- to 12-week-old female YFP-SCID mice (bred in-house). SN12^luc^ implantations were performed on 9-week-old male YFP-SCID mice. Spontaneous metastasis models involved orthotopic implantations of 2 × 10^6^ LM2-4^luc^ cells/50 μl serum-free DMEM in the right inguinal mammary fat pads of 6-week-old female CB-17 SCID mice.

### Drug preparation and *in vivo* dosing

Sunitinib malate (SU), from Pfizer or LC Laboratories, and its vehicle were formulated as described before (Ebos *et al*, 2009). SU was administered by oral gavage (PO), once daily, at a dose of 60 mg/kg mouse weight. Vasculotide (VT): VT synthesized at the Sunnybrook Research Institute (Toronto, ON, Canada) was used in experiments described in Fig2(A–D) and Supplementary Fig S5(A and B). VT synthesized by American Peptide (Sunnyvale, CA, USA) was used in experiments described in Fig5. VT synthesized by Bachem UK Ltd was used in experiments described in Figs1, 2(E–H), 3, 4, 6–9, Supplementary Figs S2, and S5(C and D). Lyophilized product was reconstituted in PBS to 500 μg/500 μl stock aliquots and stored at −80°C. As needed, stock aliquots were diluted with PBS to a 250 ng/50 μl working concentration and stored in 0.4–1 ml aliquots at −80°C. VT (at a standard dose of 250 ng/mouse) or its vehicle (PBS) was administered to mice by intraperitoneal (IP) injections every other day.

### Statistical analysis

GraphPad Prism software (San Diego, CA, USA) and Microsoft Excel were used for statistical analysis. Kaplan–Meier survival curves were compared by either the log-rank test (*P*_log-rank_) when evaluating chronic therapies or the Gehan–Breslow–Wilcoxon test (*P*_GBW_) when evaluating the long-term effects of transient/terminated therapies. Bioluminescent fluxes were log-transformed prior to comparison by Mann–Whitney tests (two-sampled) or Kruskal–Wallis tests (with Dunn's post-tests on planned comparisons) for non-Gaussian distributions, and *t-*tests (two-sampled) or one-way ANOVA (with Bonferroni's post-tests on planned comparisons) for Gaussian distributions. For all other results, see figure legends for specific statistical tests used.

## Supporting Information

Supplementary Figures S1–S6 MatMethods

Review Process File
