# Supplementary material for: Vasculotide reduces endothelial permeability and tumor cell extravasation in the absence of binding to or agonistic activation of Tie2
Source: EMBO Mol Med. 2015 Apr 7;7(6):770–87. doi: 10.15252/emmm.201404193 (PMC4459817; doi:10.15252/emmm.201404193)
Supplement: Supplementary file 1 [file emmm0007-0770-sd1.pdf]

Supplementary Information

Vasculotide reduces endothelial permeability and tumor cell extravasation in the absence of binding to or agonistic activation of Tie2

Florence T.H. Wu[1,2], Christina R. Lee[2], Elena Bogdanovic[2], Aaron Prodeus[1,3], Jean Gariépy[1,3] and Robert S. Kerbel[1,2]*

[1] Department of Medical Biophysics, University of Toronto, Toronto, ON, Canada
[2] Biological Sciences Platform, Sunnybrook Research Institute, Toronto, ON, Canada
[3] Physical Sciences Platform, Sunnybrook Research Institute, Toronto, ON, Canada
*corresponding author

Table of Contents — Page

Supplemental Methods

1. Tumor cell (TC) lines 2
2. Endothelial cells (ECs) 2
3. *In vitro* MTS cell viability assays 2
4. Generation of tumor cell-conditioned media (TC-CM) and cytokine measurements 2
5. Bioluminescence imaging (BI) of tumor burden 3
6. Immunohistochemical (IHC) analysis of lung metastases 3
7. Histological assessment of primary tumor viability and hypoxia 3
8. Western immunoblotting for total or phosphorylated Tie2 3
9. Tie2 Receptor-Binding Assays 4
References 5

Supplemental Figures

Figure S1. Structural formulas for Vasculotide and its components. 6
Figure S2. Vasculotide has no direct effect on the proliferation of Tie2^{-} tumor cells and does not reduce the inhibitory effects of sunitinib on Tie2^{+} endothelial cell viability *in vitro*. 7
Figure S3. Binding to mouse Tie2-Fc by surface plasmon resonance (SPR). 8
Figure S4. Outlines of modified Boyden chamber experiments. 9
Figure S5. Repeats of experiments where LM2-4^{luc} breast cancer cells were implanted intravenously into SCID mice. 10
Figure S6. Illustrated routes and organ tropism of *in vivo* metastatic dissemination. 11

Supplemental Methods

1. Tumor cell (TC) lines

TCs were cultured in DMEM High Glucose basal media supplemented with 5% FBS and grown humidified incubators at 37°C, 21%O_2 and 5%CO_2 unless otherwise specified. **LM2-4^{luc}** is a highly metastatically aggressive variant of the human breast carcinoma MDA-MB-231 cell line (derived through serial *in vivo* selection of lung metastases from orthotopic implantations in immunodeficient mice) that has been transfected with a firefly luciferase vector (Man et al, 2007; Ebos et al, 2009). **HT29^{luc}** is a human colon carcinoma cell line also transfected with the luciferase gene (Hackl et al, 2013). **SN12^{luc}** refers to a luciferase-tagged and highly metastatic clone of a human renal cell carcinoma cell line, SN12-PM6-L1^{luc} (Jedeszko et al, 2015). **MeWo** is a human melanoma cell line (Ebos et al, 2009).

2. Endothelial cells (ECs)

ECs were grown in humidified incubators at 37°C, 21%O_2 and 5%CO_2 unless otherwise specified. Primary human microvascular ECs derived from adult lung blood vessels (**HMVEC-LBl**, Lonza CC-2815 or **HLMEC**) or neonatal dermal blood vessels (**HMVEC-DBl**, Lonza CC-2813 or **HDMEC**) were grown in EBM-2 basal media (Lonza CC-3156), supplemented with EGM-2-MV growth factors (Lonza CC-4147, which includes 5% FBS and 2 ng/mL VEGF). Primary human umbilical vein ECs (**HUVECs**) were grown on 1% gelatinized plates in F12K media, supplemented with 10% FBS, 0.1 mg/mL heparin sodium, 10 ng/mL EGF, 10 ng/mL VEGF, 5 ng/mL bFGF and penicillin + streptomycin + 2mM L-glutamine (P/S/Glut). Transformed human **EA.hy926** and murine **MS1** ECs were grown in DMEM + 10% FBS + P/S/Glut (+ HAT for EA.hy926).

3. *In vitro* MTS cell viability assays

5,000 LM2-4^{luc} or HMVEC-LBl cells, in 100uL of their regular growth media, were seeded per well on a 96-well plate. After 18h in the cell incubator to allow for settling and plate adhesion, cells were serum-starved for 6h at 0.5% FBS to synchronize cell cycles. Cells were then treated for 48h in 150uL of either 'normal' media (basal media + 5% FBS + no other regular supplements + treatment) or 'VEGF-rich' media (basal media + 0.5% FBS + 50ng/mL VEGF + treatment). PBS and recombinant human Ang1 were used as negative and positive controls for VT treatment. Sunitinib and paclitaxel treatments were given at predetermined IC_{50} concentrations based on dose-response curves. Post-treatment, MTS/PMS solutions were prepared fresh and applied for 4h at 37°C (Promega G5421 protocol). Absorbances were read at 490nm and background-corrected with reference 560nm readings or acellular treatment-only controls.

4. Generation of tumor cell-conditioned media (TC-CM) and cytokine measurements

TC-CM were generated by first culturing LM2-4^{luc}, SN12^{luc}, or HT29^{luc} cells in DMEM+5%FBS under 21%O_2 to ~50% cell confluency. The old media were then replaced with 5mL of supplement-reduced EC growth media ("EGM/5" = 80% EBM-2 + 20% EGM-2-MV) per 10-cm dish. These dishes of TCs (along with 10-cm dishes containing only 5mL of EGM/5, without TCs, to generate the "control media") were then incubated for 30h under hypoxic conditions (1%O_2) – after which ~1.5mL of the media would have evaporated and TCs would have reached ~100% confluency with <5% apoptosis. Supernatants were then passed through 0.22μm filters to ensure that no TCs were collected with the TC-CM. Protein concentrations in TC-CM vs. control media were measured using: a Quantikine ELISA kit (R&D Systems DVE00) for human VEGF; a FlowCytomix Multiplex kit (eBioscience BMS810FF) for human IL-6 and IL-8; and a BCA protein assay kit (Thermo Scientific) for total protein.

5. Bioluminescence imaging (BI) of tumor burden

For ***in vivo* BI**: Mice were injected intraperitoneally with 150mg/kg luciferin (Promega) 10 minutes prior to and anesthetized with isofluorane just before luminescent pseudocolor imaging (uniform settings: CCD camera locked at -105°C, exposure=60s, f/1, subject size=1.5cm, bin=8, FOV=23.2cm, full image correction) using the IVIS 200 Imaging System (Caliper/PerkinElmer). Using the Living Image software (v2.50.1, Xenogen), weekly images were analyzed under uniform color range on grayscale photographic overlay. Total fluxes (photons/sec) were quantified within consistent regions of interest and statistically compared ideally at a timepoint before any deaths have occurred.

For ***ex vivo* BI:** Mice were sacrificed at 7 mins after luciferin injections, then organs of interest were dissected and individually placed within 24-well plates for imaging at 10 min post-luciferin injection.

6. Immunohistochemical (IHC) analysis of lung metastases

Surgically dissected lungs were fixed in 4% paraformaldehyde (PFA) overnight and stored in 70% ethanol. Three paraffin-embedded cross-sections per lung, 5-µm thick, 60 µm apart, were subjected to IHC staining using a serum-free protein block (Dako X0909), an anti-human vimentin antibody (clone V9, Invitrogen 18-0052; 1:100 in Dako S3022 diluent), the universal 'LSAB+, HRP' detection kit (Dako K0690), the DAB+ chromogen-substrate system (Dako K3467) and hematoxylin counterstain. Quantification procedure involved visual survey of entire tissue sections and tallying of DAB-stained metastatic nodules sizes (# cells) at 20-40X on a Leica DM LB2 Microscope. Nodule areas were then unit converted (to μm^2) and normalized to total lung section area (including air spaces) using ROI measurements from ImageJ software (NIH).

7. Histological assessment of primary tumor viability and hypoxia

5-µm thick sections of PFA-fixed paraffin-embedded mammary fat pad tumors were subjected to: (i) standard hematoxylin and eosin (H&E) staining to visualize viable vs. necrotic regions; or (ii) standard IHC staining with a goat anti-human carbonic anhydrase IX (CAIX or CA9) antibody (R&D Systems AF2188, 1:80 dilution) plus hematoxylin counterstain to visualize hypoxic regions. 2.5X images were taken on the Leica DM LB2 Microscope at fixed exposure time, saturation, γ and gain settings, then analysed on ImageJ. Under H&E, the viable periphery of each tumor can be seen as a darker cell-dense rim surrounding a lighter necrotic core – the thickness of this viable periphery was averaged over 36 measurements, spaced 10° apart around the tumor's centre (see Fig. 5D: *blue lines* on the stitched mosaic images of primary tumor cross-sections). IHC staining for CAIX further marks a hypoxic band at the edge of the viability rim bordering the necrotic core – the thickness of this hypoxic edge was averaged over 12 measurements spaced 30° apart (see Fig. 5E).

8. Western immunoblotting for total or phosphorylated Tie2

Cultured ECs and TCs were lysed in RIPA lysis buffer as previously described (Bogdanovic et al, 2006). Lung/ liver/ kidney tissues, which had been snap-frozen in liquid nitrogen once harvested from mice, were homogenized in RIPA lysis buffer (50 mM Tris-HCl pH 7.5, 150 mM NaCl, 1% Igepal, 0.5% sodium deoxycholate, 0.1% SDS, 1 mM Na_3VO_4 and protease inhibitors) at 4°C. After 1h of incubation on ice, tissue homogenates were centrifuged at 14,000 g for 5 min at 4°C, after which supernatants were recovered.

For assessment of **Tie2 receptor expression**: whole cell lysates were directly subjected to SDS-PAGE (7.5% gel), and proteins transferred to PVDF membranes were immunoblotted with an anti-Tie2 antibody (clone 33, BD Pharmingen 557039) vs. an anti-β-actin antibody (clone AC-15) (Bogdanovic et al, 2006).

For assessment of **Tie2 receptor phosphorylation**: Tie2 first had to be immunoprecipitated from cell lysates or tissue homogenates, using 3μg/15μL of an anti-Tie2 antibody (C-20, Santa Cruz sc-324) pre-coupled to 50uL of a 50% protein-A-sepharose bead slurry. To elute the immunoprecipitated Tie2, beads were then washed three times in RIPA lysis buffer, resuspended in 85μL SDS sample buffer and boiled for 10 mins. To avoid stripping-and-reprobing, supernatants were divided and subjected to PAGE in parallel as follows: 60 μL for the detection of phosphorylated Tie2 and 20 μL for the assessment of total Tie2. Transferred Tie2 proteins on the parallel PVDF membranes were immunoblotted with antibodies for phosphotyrosine (clone 4G10, Millipore 05-321) and total Tie2 (clone 33, BD Pharmingen 557039) respectively (Bogdanovic et al, 2006).

Semi-quantitative densitometry analysis of immunoblots was performed using the ChemiGenius2 bioimaging system and GeneSnap/GeneTools software (Syngene).

9. Tie2 Receptor-Binding Assays

Vasculotide (VT) was manufactured by Bachem UK Ltd. **T7c** (CHHHRHSF) was synthesized by Genscript (Piscataway, NJ, USA). **PEG-Cys** consists of a single cysteine reacted to each of the 4 arms of PEG-maleimide (Sunbright PTE-100MA, NOF Corporation, Tokyo, Japan). **BowAng1** (Ang1-Fd-Fc-Fd) was made and provided by Regeneron Pharmaceuticals, Inc. (Tarrytown, NY, USA). **COMP-Ang1** was purified in the Kerbel lab from serum-free culture media conditioned by an engineered CHO cell line provided by Dr. Gou Young Koh (KAIST), according to published protocols (Hwang et al, 2008). Recombinant human Angiopoietin-1 (**rhAng1**) and Vascular Endothelial Growth Factor (**rhVEGF**) were purchased from R&D Systems. Recombinant human and mouse Tie2 Fc chimera were purchased from R&D Systems (**hTie2-Fc**: 313-TI-100; **mTie2-Fc**: 762-T2-100).

Pull-Down Assay: rhAng1 (20ng) or VT (100ng) was incubated with 0 or 150ng of m/hTie2-Fc in 500μL of buffer solution (50mM Tris, 100mM NaCl, 0.05% CHAPS*, pH 7.4) at 4°C for 2h on a nutator. In parallel, a 50% protein A sepharose bead slurry was pre-blocked 2:1 with 3% BSA at 4°C for 2h on a nutator. 72μL of the pre-blocked protein A sepharose beads was then mixed into each sample and incubated at 4°C for another 1.5h on a nutator. Bead-conjugated samples were washed three times with 1mL of buffer solution. Supernatants were kept for the assessment of residual ligand unbound to bead-conjugated Tie2-Fc. To elute bound ligands, beads were boiled at 100°C for 5min in SDS sample buffer, with β-mercaptoethanol (β-ME) for Ang1 blot samples, and without β-ME for VT blot samples. The controls – 20ng of rhAng1 (total input), 100ng of VT (total input), 73.6ng of PEG-Cys (structural/molecular weight control for VT), and 40 μL of supernatant (7% of residual/unbound ligand) – were similarly boiled in SDS sample buffer. Ang1 samples and controls were resolved by 7.5% polyacrylamide gel electrophoresis (PAGE), electroblotted onto PVDF membranes, and probed with an anti-Ang1 antibody (R&D Sytems, AF923; diluted 1:2,000 in TBS supplemented with 7% BSA and 0.1% Tween-20) at 75kDa (reduced monomers). VT samples and controls were resolved by 17% PAGE, blotted onto PVDF membranes, and probed with an anti-PEG antibody (Silver Lake Research, CH2074; diluted 1:4,000 in TBS supplemented with 7% BSA and 0.05% CHAPS*) at higher "apparent" molecular weights than the expected 14 and 10kDa due to their polymeric nature and large hydrodynamic radii (Zheng et al, 2007). *Note: non-ionic, PEO-based, polymer detergents were avoided at these steps as they cross-react with the anti-PEG antibody used for detecting VT.

Surface Plasmon Resonance (SPR) Experiments: The binding of analytes – VT, T7c peptide, PEG-Cys, rhAng1, COMP-Ang1, BowAng1, and rhVEGF – to the extracellular domain of Tie2 were

investigated by SPR using a Biacore T200 Instrument (GE Healthcare). For qualitative binding analysis, mTie2-Fc was dissolved in 10mM acetate buffer (pH 4.5) and immobilized at high density onto a CM5 sensor chip using amine coupling chemistry as described by the manufacturer to a response of 11,500RU. Binding analysis were performed by exposure of immobilized mTie2-Fc to the analytes diluted in HBS-EP (0.01M HEPES pH 7.4, 0.15M NaCl, 3mM EDTA, 0.005% v/v Tween-20), which was supplemented with 1mg/mL carboxymethyl dextran (NSB reducer, GE Healthcare) to reduce non-specific binding to the sensor chip. Analytes were injected at 30μL/min (association time 120s, dissociation time 120s) at 37°C, and the chip surface was regenerated each cycle by a 60s pulse of 4M $MgCl_2$. To quantitatively determine the kinetic properties of the interaction (K_D, k_d, k_a) and avoid mass transport limitations, mTie2-Fc and hTie2-Fc were immobilized at low density (1,000-1,200RU) onto separate flow cells of the CM5 chip using amine coupling chemistry. Kinetic analyses were performed using a single-cycle kinetic procedure where immobilized Tie2-Fc was exposed to increasing concentrations (0.625-10 nM) of analyte dissolved in HBS-EP with 1mg/ml carboxymethyl dextran. Kinetic experiments were performed at 37°C with a flow rate of 30μL/min (association time 120s, dissociation time 120s) and the chip surface regenerated each cycle with 4M $MgCl_2$. Sensorgrams were double reference subtracted against responses to a Tie2-free reference flow cell and buffer-only injections. All data were analysed using BIA Evaluation Software 2.0 (GE Healthcare) to calculate k_d, k_a and K_D using the 1:1 ligand binding model. Chi^2 and residual values were subsequently used to confirm quality of fit between experimental and modeled data sets.

REFERENCES

Bogdanovic E, Nguyen VP, Dumont DJ (2006) Activation of Tie2 by angiopoietin-1 and angiopoietin-2 results in their release and receptor internalization. J Cell Sci 119: 3551-3560.

Ebos JM, Lee CR, Cruz-Munoz W, Bjarnason GA, Christensen JG, Kerbel RS (2009) Accelerated metastasis after short-term treatment with a potent inhibitor of tumor angiogenesis. Cancer Cell 15: 232-239.

Hackl C, Man S, Francia G, Milsom C, Xu P, Kerbel RS (2013) Metronomic oral topotecan prolongs survival and reduces liver metastasis in improved preclinical orthotopic and adjuvant therapy colon cancer models. Gut 62: 259-271.

Hwang SJ, Kim SH, Kim HZ, Steinmetz MO, Koh GY, Lee GM (2008) High-level expression and purification of a designed angiopoietin-1 chimeric protein, COMP-Ang1, produced in Chinese hamster ovary cells. Protein J 27: 319-326.

Jedeszko C, Paez-Ribes M, Di Desidero T, Man S, Lee CR, Xu P, Bjarnason GA, Bocci G, Kerbel RS (2015) Postsurgical Adjuvant or Metastatic Renal Cell Carcinoma Therapy Models Reveal Potent Anti-Tumor Activity of Metronomic Oral Topotecan with Pazopanib. Sci Transl Med [In press].

Man S, Munoz R, Kerbel RS (2007) On the development of models in mice of advanced visceral metastatic disease for anti-cancer drug testing. Cancer Metastasis Rev 26: 737-747.

Zheng C, Ma G, Su Z (2007) Native PAGE eliminates the problem of PEG-SDS interaction in SDS-PAGE and provides an alternative to HPLC in characterization of protein PEGylation. Electrophoresis 28: 2801-2807.

Supplemental Figures

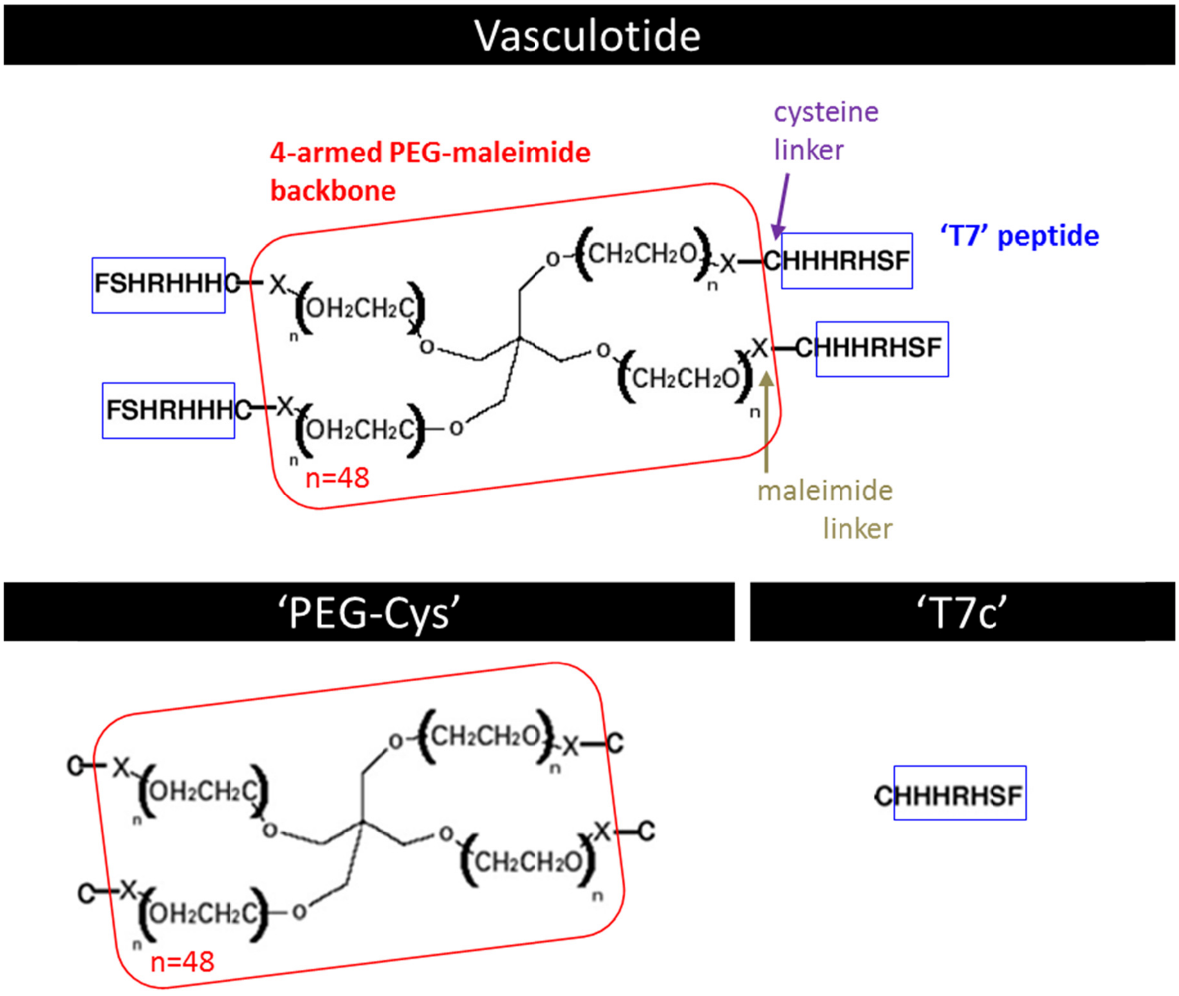

Figure S1. Structural formulas for Vasculotide and its components.

Each molecule of Vasculotide consists of four 'T7' peptides (HHHRHSF; Tournaire et al. *EMBO rep.* 2004; 5(3): 262-267.) bonded via a cysteine to the maleimide functional groups of a four-armed PEG backbone (Sunbright PTE-100 MA, NOF America Corporation). 'PEG-Cys' (~10.5kDa) and 'T7c' (~1kDa) were used as structural controls in modified Boyden chamber experiments.

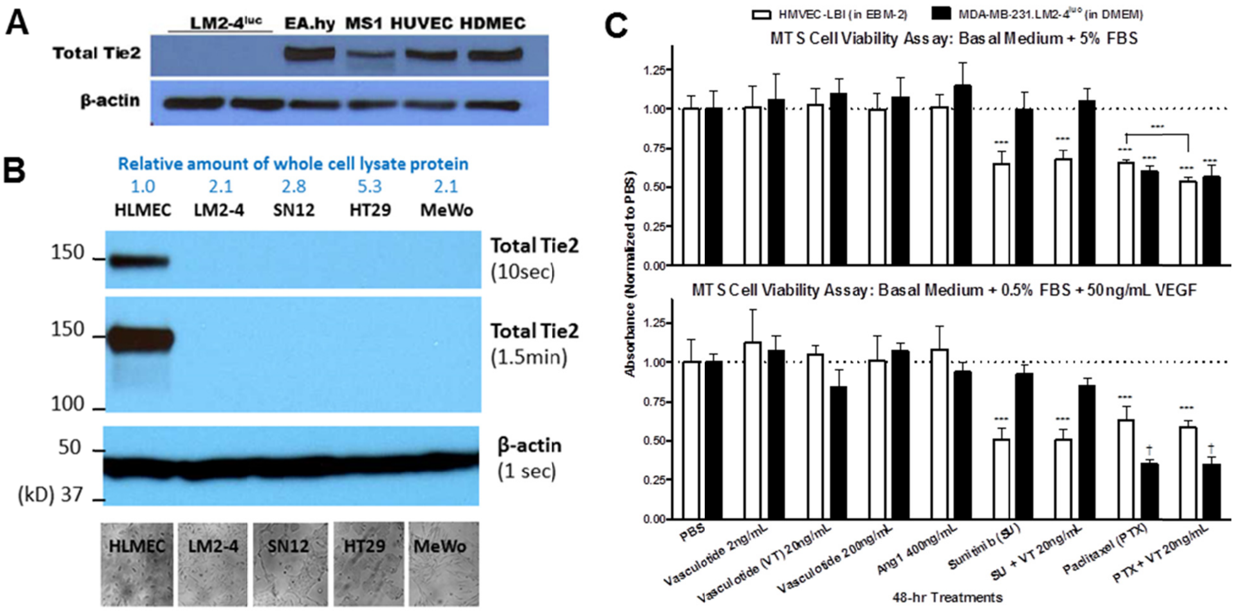

Figure S2. Vasculotide has no direct effect on the proliferation of Tie2^{-} tumor cells and does not reduce the inhibitory effects of sunitinib on Tie2^{+} endothelial cell viability *in vitro*.

A-B: Western immunoblotting of whole cell lysates showed abundant protein expression of Tie2 on different endothelial cell populations (EA.hy926, MS1, HUVEC, HDMEC, HLMEC) and undetectable levels within highly concentrated tumor cell lysates (breast LM2-4^{luc}, renal SN12^{luc}, colon HT29^{luc} and melanoma MeWo). β-actin served as loading controls. **C:** MTS assays measured the effects of 48-hour treatments on the viability of endothelial cells (HMVEC-LBl) and tumor cells (LM2-4^{luc}) cultured with 'normal' (5% FBS) or 'VEGF-rich' (0.5% FBS + 50ng/mL VEGF) supplementation. 400ng/mL Ang1 is the estimated equimolar control for 20ng/mL VT treatment. Sunitinib (SU) – given at its IC_{50} of 2μM and 5μM under 'normal' and 'VEGF-rich' culturing conditions respectively – is a prototypical VEGF pathway-targeting antiangiogenic TKI, which preferentially inhibited the survival and growth of ECs, compared to TCs. Paclitaxel (PTX) – given at an IC_{50} of 500nM – is a prototypical chemotherapeutic agent, which preferentially inhibited mitosis in highly proliferative TCs, compared to ECs, under 'VEGF-rich' conditions. In contrast to SU or PTX, 48-hour VT treatments ranging from 0.1ng/mL to 1000ng/mL did not significantly alter the viability of either ECs or TCs. Importantly, the presence of VT also did not interfere with SU inhibition of EC viability or PTX inhibition of TC viability. Means ± SD are shown. Two-sampled *t*-tests (n=5-6): *** $P < 0.001$; † $P <$ 1e-8; comparisons with PBS unless otherwise noted; same trends reproduced in duplicate experiments.

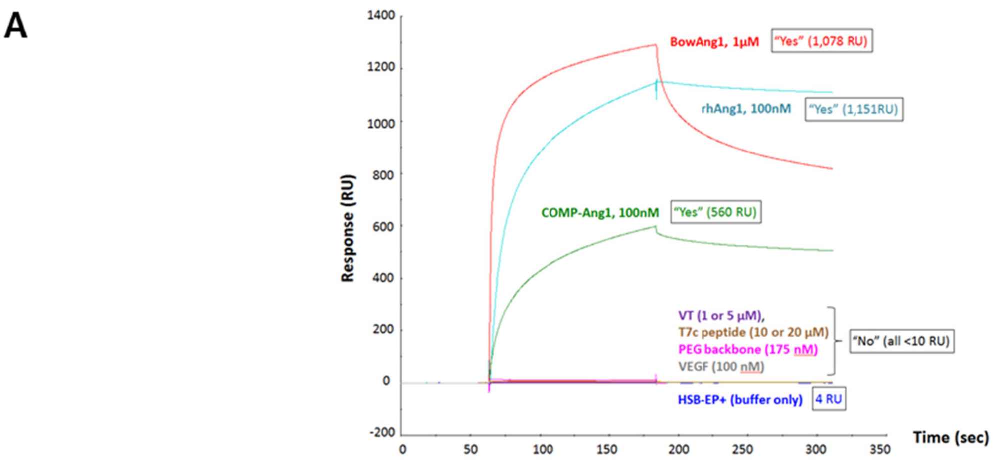

B

SPR: Binding to MouseTie2-Fc

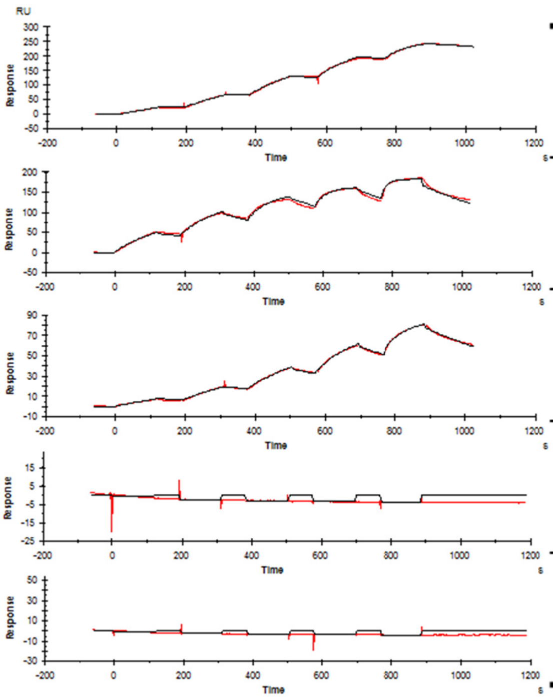

	On-rate, K_a (1/Ms)	Off-rate, K_d (1/s)	Dissociation constant, K_D
rhAng1	1.393×10^6	0.00033	0.237 nM
BowAng1	4.973×10^6	0.00231	0.465 nM
COMP-Ang1	1.574×10^6	0.00208	1.319 nM
Vasculotide	Error: Kinetic constants cannot be uniquely determined.		
rhVEGF	Error: Kinetic constants cannot be uniquely determined.		

Figure S3. Binding to mouse Tie2-Fc by surface plasmon resonance (SPR).

A: Qualitative runs of various analytes, at high concentrations (not molar-equivalent), over high-density mouse Tie2-Fc. The Ang1 variants – rhAng1, BowAng1, COMP-Ang1 – all strongly bound mTie2-Fc. In contrast, there was no detectable binding of VT, PEG-Cys, T7c, or VEGF to mTie2-Fc compared to the buffer-only control. **B**: Quantitative analysis of various analytes, at physiologically-relevant concentrations (0.625 to 10nM) and conditions (37°C and pH 7.4), over low-density mouse Tie2-Fc. This panel shows the mouse equivalent of the human Tie2-Fc experiment summarized in ***Fig. 9C***.

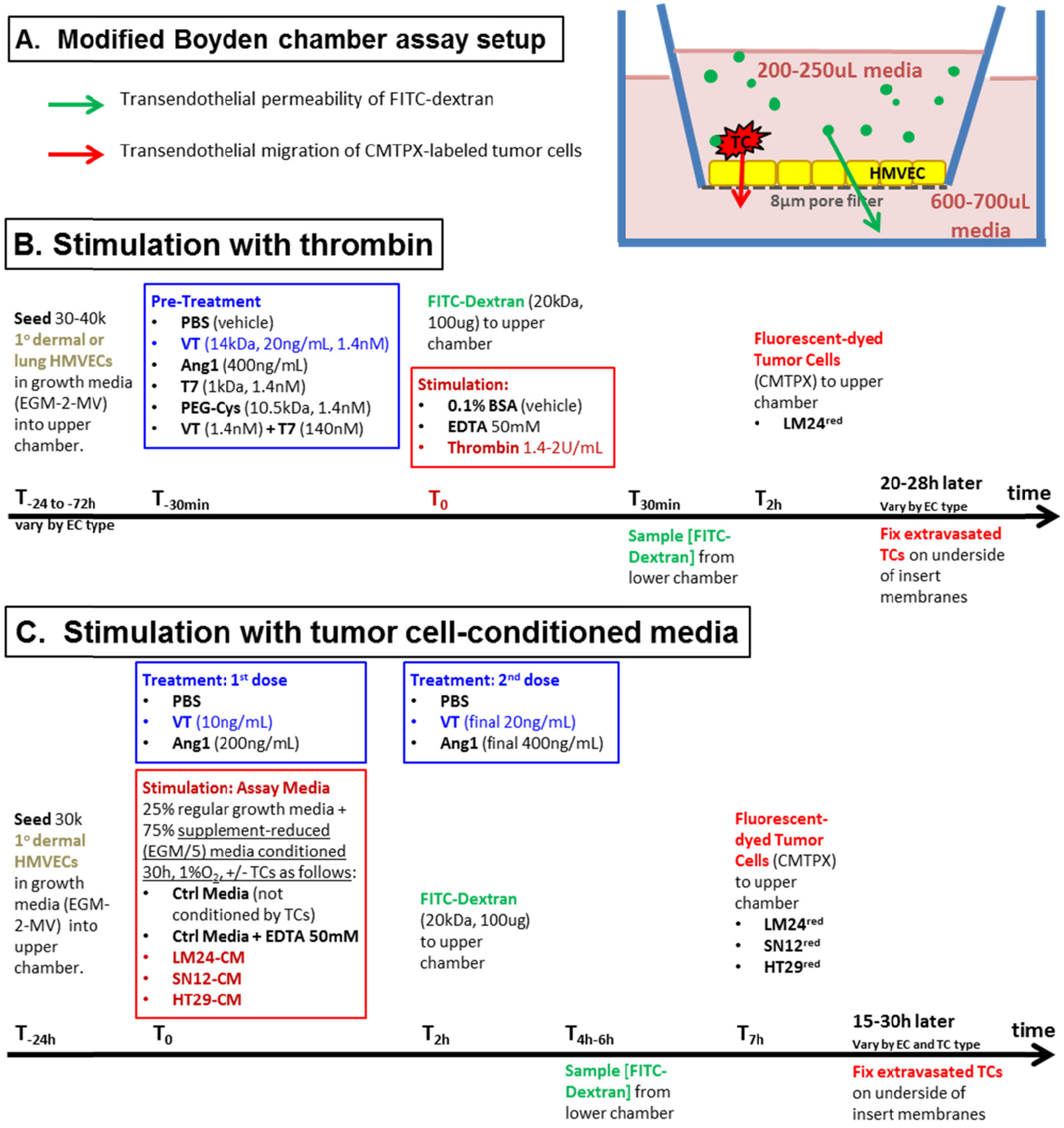

Figure S4. Outlines of modified Boyden chamber experiments.

A: Illustration of Boyden chamber setup. **B**: A typical "thrombin stimulation" experiment, where treatment with Vasculotide (VT) or Ang1 begins 30mins prior to fast-acting thrombin stimulation of endothelial cells (ECs). **C**: A typical "tumor cell-conditioned media (TC-CM)" experiment, where the first of two doses of VT or Ang1 is administered at the same that ECs are exposed to slower-acting TC-CM.

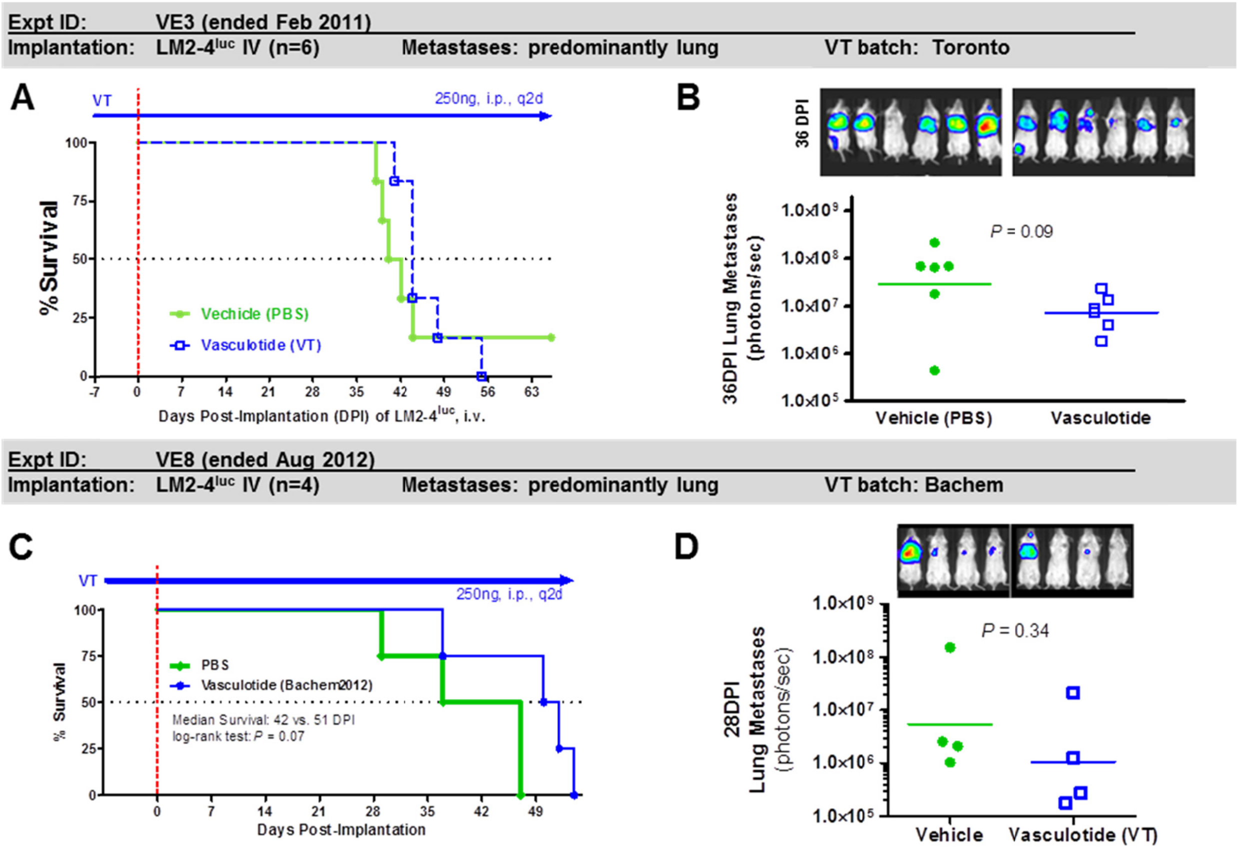

Figure S5. Repeats of experiments where LM2-4^{luc} breast cancer cells were implanted intravenously into SCID mice.

Two repeat experiments where Vasculotide monotherapy demonstrated the same trends of inhibiting lung metastases – reducing bioluminescent activity (**B** and **D**; consistent with ***Fig. 2D***) and prolonging median survival as limited by lung metastases (**A** and **C**; consistent with ***Fig. 2A***). Geometric means and *P* values derived from Mann-Whitney tests are depicted in **B** and **D**.

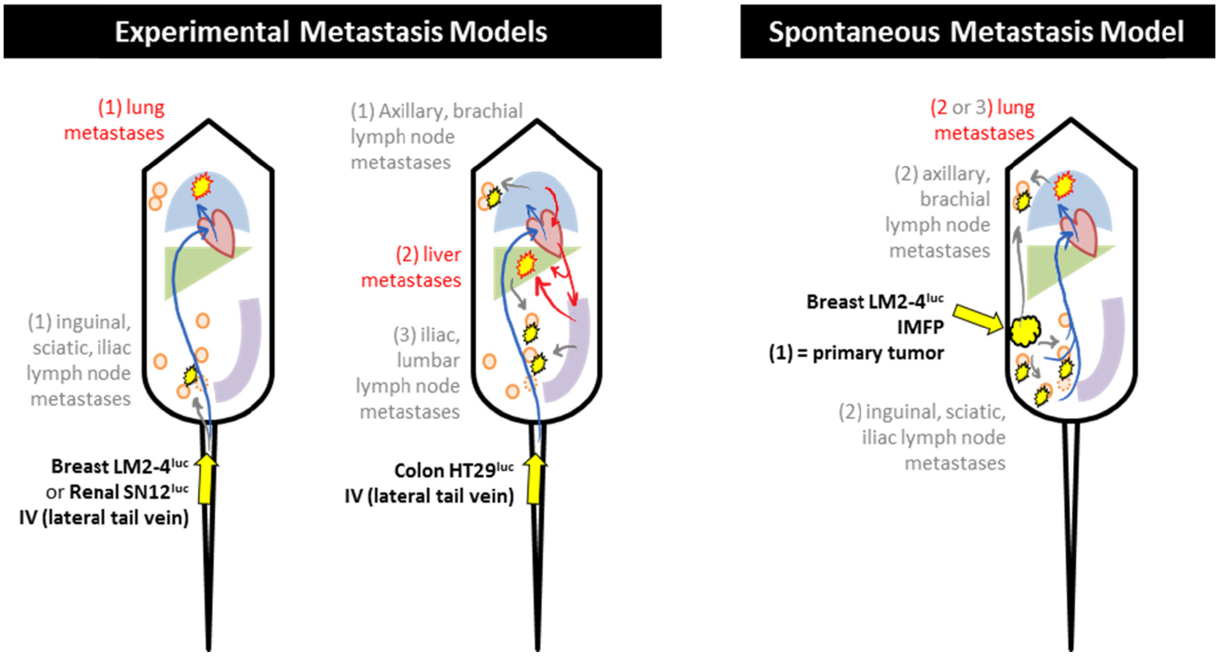

Figure S6. Illustrated routes and organ tropism of *in vivo* metastatic dissemination.

The likely pathways of metastatic dissemination are illustrated on schematic representations of mouse organs (lungs in *blue*, heart in *red*, liver in *green*, intestines in *purple* and lymph nodes in *orange*). *Bold yellow arrows* mark the point of tumor cell implantations. *Thin arrows* indicate the direction of transit through lymphatic circulation in *grey* and blood circulation in *blue/red*. *Yellow clouds* represent primary tumor or metastatic growths, with associated text descriptions – *grey* text for lymphatic/lymphogenous metastases (developing after intravasation into lymphatic capillaries) and *red* text for haematogenous metastases (developing after extravasation across blood capillaries). Numbers indicate the temporal order of tumor burden development and metastatic seeding.
