# Supplementary material for: Vasculotide reduces endothelial permeability and tumor cell extravasation in the absence of binding to or agonistic activation of Tie2
Source: EMBO Mol Med. 2015 Apr 7;7(6):770–87. doi: 10.15252/emmm.201404193 (PMC4459817; doi:10.15252/emmm.201404193)
Supplement: Supplementary file 2 [file emmm0007-0770-sd2.pdf]

Vasculotide reduces endothelial permeability and tumor cell extravasation in the absence of binding to or agonistic activation of Tie2

Florence T. H. Wu, Christina R. Lee, Elena Bogdanovic, Aaron Prodeus, Jean Gariépy and Robert S. Kerbel

Corresponding author: Robert S. Kerbel, University of Toronto, Sunnybrook Res. Institute

Review timeline:

	Submission date:	29 April 2014
	Accepted:	09 March 2013

Transaction Report:

No Peer Review Process File is available with this article, as the authors have chosen not to make the review process public in this case.

© EMBO
