# Supplementary figures and images for: Vasculotide reduces endothelial permeability and tumor cell extravasation in the absence of binding to or agonistic activation of Tie2
Source: EMBO Mol Med. 2015 Apr 7;7(6):770–87. doi: 10.15252/emmm.201404193 (PMC4459817; doi:10.15252/emmm.201404193)

Source Data for Fig. 7A-E

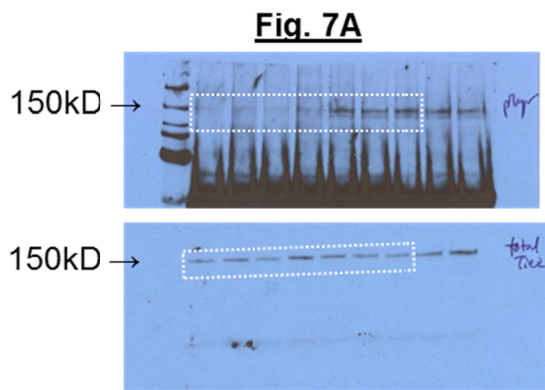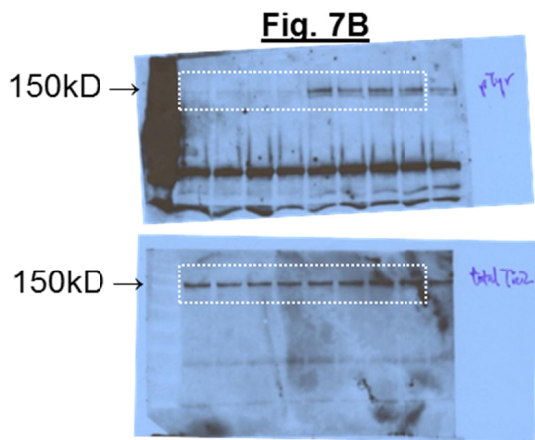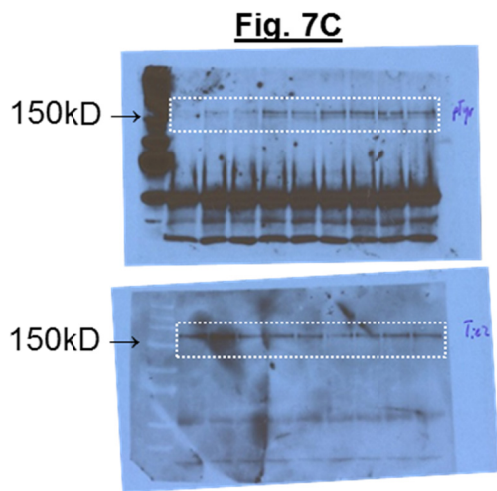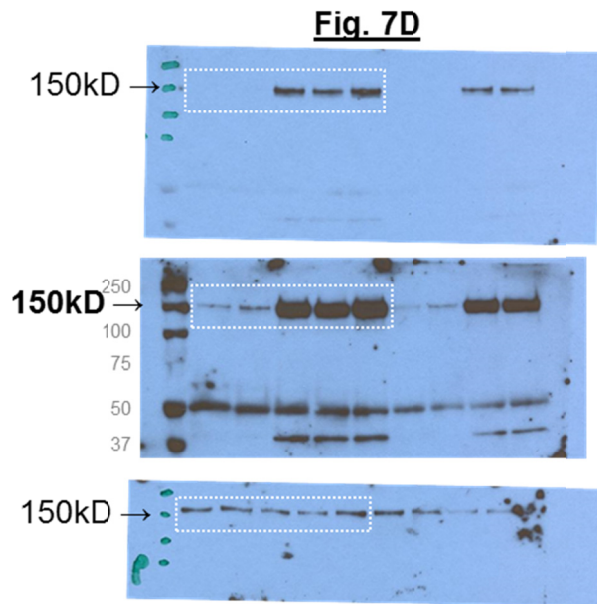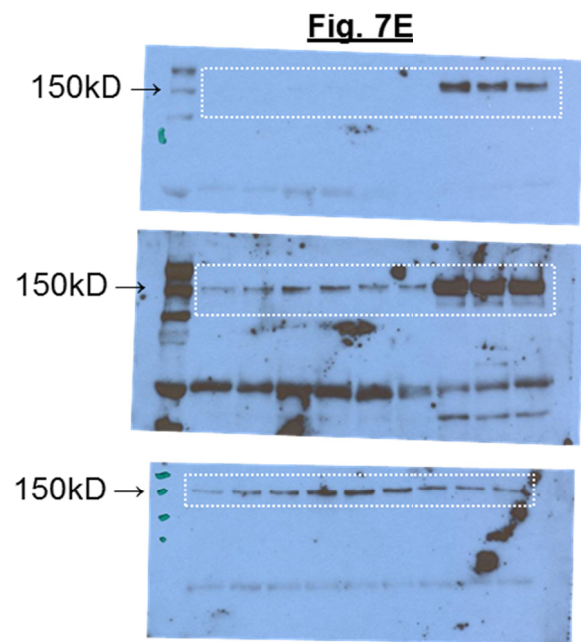

Source Data for Fig. 7F

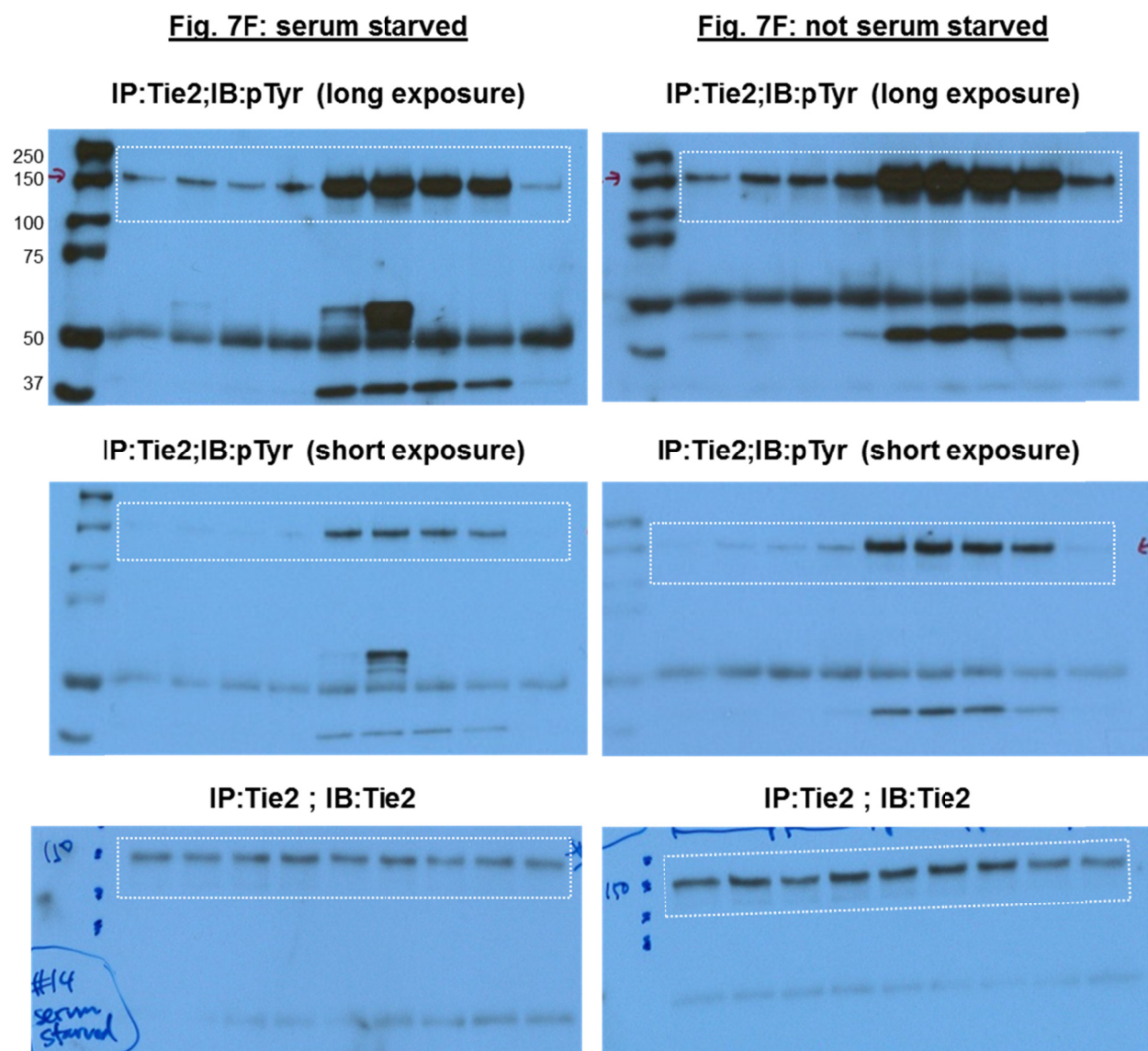

Supplement: Supplementary file 3 [file emmm0007-0770-sd3.pdf]

Source Data for Fig. 8A

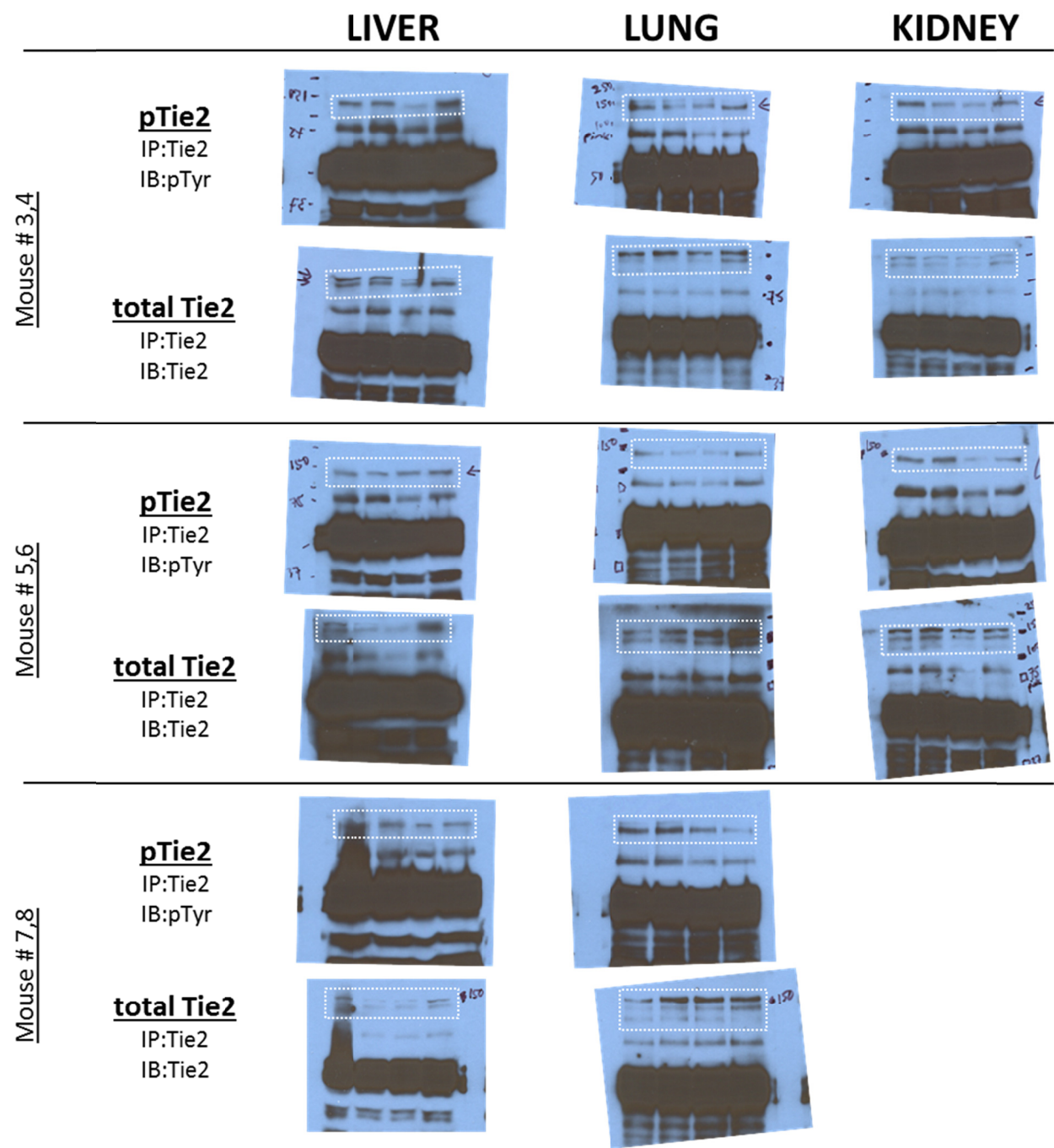

Supplement: Supplementary file 4 [file emmm0007-0770-sd4.pdf]

## Source Data for Fig. 9A-B

---

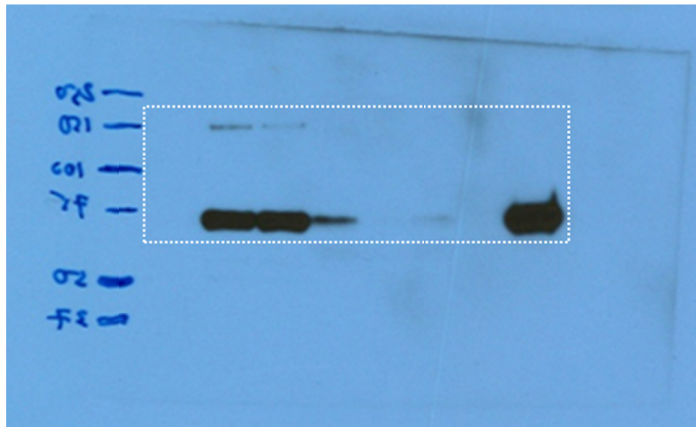

← Ang1 (Fig. 9A)

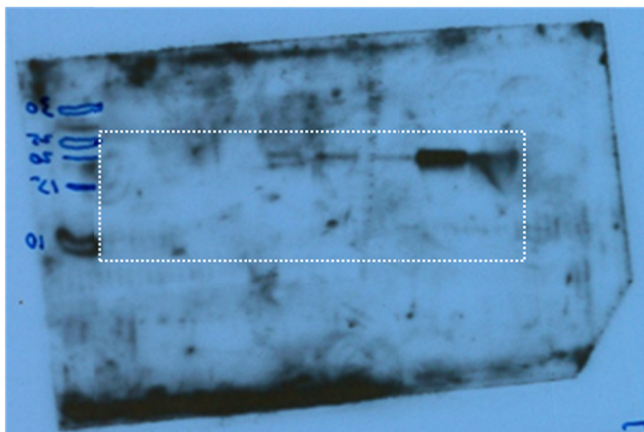

← PEG (Fig. 9B)

Supplement: Supplementary file 5 [file emmm0007-0770-sd5.pdf]
